# Development of a dual-component infection-resistant arterial replacement for small-caliber reconstructions: A proof-of-concept study

**DOI:** 10.3389/fbioe.2023.957458

**Published:** 2023-01-18

**Authors:** Sonia Zia, Adrian Djalali-Cuevas, Michael Pflaum, Jan Hegermann, Daniele Dipresa, Panagiotis Kalozoumis, Artemis Kouvaka, Karin Burgwitz, Sofia Andriopoulou, Alexandros Repanas, Fabian Will, Karsten Grote, Claudia Schrimpf, Sotiria Toumpaniari, Marc Mueller, Birgit Glasmacher, Axel Haverich, Lucrezia Morticelli, Sotirios Korossis

**Affiliations:** ^1^ Lower Saxony Centre for Biomedical Engineering Implant Research and Development, Hannover Medical School, Hannover, Germany; ^2^ Institute of Functional and Applied Anatomy, Research Core Unit Electron Microscopy, Hannover Medical School, Hannover, Germany; ^3^ Institute for Multiphase Processes, Leibniz University Hannover, Hannover, Germany; ^4^ LLS ROWIAK LaserLabSolutions GmbH, Hannover, Germany; ^5^ Cardiology and Angiology, Philipps-University Marburg, Marburg, Germany; ^6^ Department of Cardiothoracic, Transplantation and Vascular Surgery, Hannover Medical School, Hannover, Germany; ^7^ Cardiopulmonary Regenerative Engineering Group (CARE), Centre for Biological Engineering, Loughborough University, Loughborough, United Kingdom; ^8^ Wolfson School of Mechanical, Electrical and Manufacturing Engineering, Loughborough University, Loughborough, United Kingdom

**Keywords:** small-caliber vascular graft, tibial artery, decellularized scaffold, polymeric sleeve, biomechanics, antimicrobial activity

## Abstract

**Introduction:** Synthetic vascular grafts perform poorly in small-caliber (<6mm) anastomoses, due to intimal hyperplasia and thrombosis, whereas homografts are associated with limited availability and immunogenicity, and bioprostheses are prone to aneurysmal degeneration and calcification. Infection is another important limitation with vascular grafting. This study developed a dual-component graft for small-caliber reconstructions, comprising a decellularized tibial artery scaffold and an antibiotic-releasing, electrospun polycaprolactone (PCL)/polyethylene glycol (PEG) blend sleeve.

**Methods:** The study investigated the effect of nucleases, as part of the decellularization technique, and two sterilization methods (peracetic acid and γ-irradiation), on the scaffold’s biological and biomechanical integrity. It also investigated the effect of different PCL/PEG ratios on the antimicrobial, biological and biomechanical properties of the sleeves. Tibial arteries were decellularized using Triton X-100 and sodium-dodecyl-sulfate.

**Results:** The scaffolds retained the general native histoarchitecture and biomechanics but were depleted of glycosaminoglycans. Sterilization with peracetic acid depleted collagen IV and produced ultrastructural changes in the collagen and elastic fibers. The two PCL/PEG ratios used (150:50 and 100:50) demonstrated differences in the structural, biomechanical and antimicrobial properties of the sleeves. Differences in the antimicrobial activity were also found between sleeves fabricated with antibiotics supplemented in the electrospinning solution, and sleeves soaked in antibiotics.

**Discussion:** The study demonstrated the feasibility of fabricating a dual-component small-caliber graft, comprising a scaffold with sufficient biological and biomechanical functionality, and an electrospun PCL/PEG sleeve with tailored biomechanics and antibiotic release.

## 1 Introduction

The incidences of occlusive coronary and peripheral arterial disease, and lower extremity chronic deep venous insufficiency are increasing worldwide (Fowkes et al., 1991), whereas the failure rate for arterio-venous fistulas for hemodialysis is .95 fistulas per patient per year (Lemson et al., 2000). Vascular grafting is one of the most performed surgical procedures for treating atherosclerotic, aneurysmal and stenotic blood vessels. The gold standard for grafting involves the use of autologous internal thoracic artery, long saphenous vein, internal mammary artery, radial, gastroepiploic or tibial artery for coronary (Gaudino et al., 2015), infrainguinal, or other peripheral revascularization (Klinkert et al., 2004), and for the creation of arterio-venous fistulas in hemodialysis patients (Pisoni et al., 2002). However, there is no suitable autologous vessel available for grafting in 10%–40% of patients due to trauma, disease and/or previous operations (Derham et al., 2008). Even though prosthetic (synthetic) grafts, such as Dacron^®^ and expanded polytetrafluoroethylene, perform well in large diameter vessels (>6 mm), they perform poorly in low-flow small-caliber (<6 mm) anastomoses due to intermediate-term failure from intimal hyperplasia and thrombosis, caused by the lack of a thromboresistant blood-contacting surface, adverse haemodynamics and compliance mismatch between the prosthesis and the host vessel, that lead to a 10-year patency rate of approximately 50% (Lemson et al., 2000; Yow et al., 2006; Gaudino et al., 2015). Vascular homografts and bioprosthetic (chemically treated) xenografts have been used as alternatives to autologous conduits. However, these have demonstrated aneurysmal degeneration and rupture, whereas bioprostheses with cell remnants and xenoantigens will calcify, become rigid and degenerate, and homografts are associated with limited availability and immunogenicity (Dardik et al., 2002).

In contrast to prosthetic, bioprosthetic and homograft conduits that cannot regenerate in the patient, decellularized grafts have been reported to recellularize *in vitro* and *in vivo* and have the potential to remodel and regenerate (Yow et al., 2006; Derham et al., 2008; Katsimpoulas et al., 2015; Ma et al., 2017). Decellularized animal or human tissues offer natural 3D histoarchitecture and constitution, with appropriate physical and biological cues for tethering cell colonization, and reduced immunogenicity, providing that decellularization can remove xenoepitopes from animal tissues (Luo et al., 2014). Most previous studies on decellularized vascular conduits have focused on the use of carotid (Böer et al., 2013), coronary (Campbell et al., 2012), radial (Meiring et al., 2017), umbilical (Rodríguez-Rodríguez et al., 2018) and femoral (Meiring et al., 2017) arteries, iliac (Olausson et al., 2012), inferior vena cava (Teebken et al., 2000), great saphenous (Teebken et al., 2009), jugular (Mogaldea et al., 2017) and umbilical (Rodríguez-Rodríguez et al., 2018) veins, as well as abdominal aorta (Katsimpoulas et al., 2015) and ureter (Yow et al., 2006; Derham et al., 2008). To the best of the authors’ knowledge there have been no reports on the decellularization of the tibial artery. Tibial artery is widely used for lower extremity reconstructions (Kawarada et al., 2014) and presents a good-size match to coronary arteries (Benjacholamas et al., 2002; Telang et al., 2016), whereas its arterial nature makes it a favorable choice over venous counterparts for coronary reconstructions (Benjacholamas et al., 2002).

Regardless of the specific vascular condition, conventional vascular grafting is also confronted with increased susceptibility to infection, with a relatively high incidence at the implantation site. Graft infection carries a high rate of mortality and amputation, and has a reported incidence of between 1%–8%, depending on the implantation site, indication for primary intervention, underlying disease, and host defense mechanism (Young et al., 2012). Such infections are more common after emergency procedures for ruptured aneurysms and for peripheral and abdominal prosthetic graft implantation (Young et al., 2012). Systemic antibiotic therapy has been reported to be unable to reach a sufficient local concentration to suppress bacterial adhesion on the graft. In order to induce antimicrobial activity at the site of implantation, studies have reported on the pre-soaking/coating of prosthetic grafts with antimicrobial agents (Bisdas et al., 2012), fibrin sealant/antibiotic compounds (Kuehn et al., 2010) and antibiotic-releasing polymers (Grafmiller et al., 2016). However, such direct treatments on biological grafts might induce changes in their biomechanics and function and might also interfere with any potential cellularization of decellularized scaffolds *in vivo*.

This overview indicates a major clinical need for novel and smarter solutions, encompassing implants that can fully integrate and regenerate with the patient, whilst providing postoperative antimicrobial prophylaxis. The aim of the present study was to develop a dual-component graft for small-caliber arterial reconstructions, comprising a decellularized tibial artery scaffold and an antibiotic-releasing, electrospun PCL/PEG blend outer sleeve. The overarching approach of this study is presented in graphical form in the Supplementary Figure S1 in the Supplement. Although porcine tibial arteries (PTAs) were used as a model of tibial artery homografts, decellularized PTAs could be potentially used clinically, providing that they are free of immunogenic xenoepitopes. PCL is semi-crystalline highly hydrophobic polymer that has gained FDA approval for clinical use. PCL can be easily solubilized in most organic solvents and can be easily electrospun, whereas it has good miscibility and demonstrates good mechanical stability and prolonged degradation. PEG is a water-soluble biocompatible polyether with a wide range of molecular weights, which has been used in hydrogels, and as a surface modification and adhesion molecule (Repanas et al., 2015). The diverse properties of PCL and PEG make them ideal candidates for tailoring PCL/PEG blends that exhibit a combination of properties. The objectives of the study were to i) conduct an in-depth assessment of the effect of a modified version of the in-house decellularisation protocol on the morphological, biological and biomechanical properties of the produced small-caliber PTA scaffolds, ii) quantify the effect of nuclease enzymes, as part of the decellularization technique, on DNA removal, iii) assess two commonly used sterilization methods (peracetic acid and γ-irradiation) on the biological and biomechanical integrity of the PTA scaffolds, and iv) investigate two different PCL/PEG ratios on the antimicrobial, biological and biomechanical properties of the polymer blend sleeve.

## 2 Materials and methods

### 2.1 Tissue procurement, disinfection, decellularisation, and sterilization

Porcine legs were obtained from 6-month-old German Landrace pigs from the local abattoir and the PTAs were isolated within 4 h of animal slaughter. The adventitial surface of PTAs was scraped with a scalpel blade and washed in phosphate-buffered saline (PBS) to remove excess connective tissue and blood. The PTAs were tested immediately after dissection (Native), or placed in 150 mL square Schott bottles and disinfected in 100 mL of antibiotics solution [0.2 mg/mL polymyxin B sulfate, Sigma-Aldrich; .05 mg/mL vancomycin hydrochloride hydrate (VAN), Sigma-Aldrich; 0.5 mg/mL gentamicin sulfate (GEN); Biochrom] in PBS (pH7.2) at 37°C for 1 h. The PTAs were then decellularized according to a modified version of the in-house protocol (Theodoridis et al., 2016). Specifically, the PTAs were placed in 150 mL square Schott bottles and treated 2 × 12 h with hypotonic buffer (10 mM Tris, 2.7 mM EDTA), before they were treated 2 × 12 h with .5% (w/v) Triton X-100 (Roth) in distilled water (DW) and then 2 × 12 h with .5% (w/v) sodium dodecyl sulfate (SDS; Roth) in DW, at room temperature (RT). The samples were then rinsed 6 × 12 h in PBS. Following decellularization, the PTA scaffolds were split into two groups. The first group (method 1; M1) was sterilized straight after decellularization by either immersion in .1% (v/v) peracetic acid (PAA; Sigma-Aldrich) in PBS (pH7.3) for 3 h, followed by 3 × 30 min washes in PBS (M1-PAA) (Morticelli et al., 2013; Granados et al., 2017), or by 150 Gy of γ-irradiation (M1-γ), and stored in PBS at 4°C until testing. The second group (method 2; M2) was incubated with nucleic acid digestion solution (50.35 mM Tris, 21 mM MgCl2, pH 7.5–7.7), containing 50 U/mL DNase (Sigma-Aldrich) and 1 U/mL RNase (Sigma-Aldrich) for 3 h at 37°C, followed by 3 × 30 min washes in PBS. The PTAs were then sterilized with PAA (M2-PAA) or γ-irradiation (M2-γ), and stored in PBS at 4°C until testing. All decellularization and sterilization steps were carried out under aseptic conditions and agitation (180 rpm) in an elliptical shaker (GFL 3031), whereas all solutions were autoclaved or filtered sterilized. The sterility of the treated PTAs was assessed by incubating 24 mL of the final washing solution with 6 mL of Soy-Casein medium (Roth X938.1) in filter-cap falcon tubes for 14 days at 37°C. The negative control was 24 mL of sterile PBS incubated with 6 mL Soy-Casein medium. Contamination was assessed by the medium turbidity; contaminated samples were eliminated from further assessment.

### 2.2 Polymer sleeve fabrication and sterilization

Tubular polymer blend sleeves with two different PCL/PEG ratios, with or without antibiotics, were fabricated by electrospinning. Polymer blend solutions were prepared by dissolving PCL (Mn 70,000–90000; Sigma-Aldrich) and PEG (Mr 3,500–4,500; Fluka) in 2,2,2-trifluroethanol (TFE; abcr GmbH) under constant stirring (300 rpm) for 18 h at RT, in order to achieve homogenous mixtures. The total polymer concentration was either 200 mg/mL (150 mg/mL PCL and 50 mg/mL PEG; group 150:50) or 150 mg/mL (100 mg/mL PCL and 50 mg/mL PEG; group 100:50), rendering PCL/PEG mass ratios of 3:1 and 2:1, respectively. The concentrations of PCL and PEG were selected based on previous studies (Repanas et al., 2015; Repanas et al., 2016) and preliminary experiments, considering the ease of electrospinning of the solutions and the solubility of the polymers in TFE. In addition, solutions with the same polymer concentrations and mass ratios, but supplemented with 5 mg/mL VAN and 5 mg/mL GEN were also prepared (groups 150:50A and 100:50A). Electrospinning was conducted according to Repanas et al. (2015), Repanas et al. (2016). Specifically, the polymer blend solutions were transferred to 10 mL syringes (Omnifix), which were fitted with disposable blunt-tipped 0.6 mm-inner-diameter needles (21G Nordson EFD) and used as reservoirs. The flow rate of the solution was maintained at 4 mL/h with an applied voltage of 25 kV, whereas the distance between the needle tip and the grounded cylindrical collector was set at 25 cm. During electrospinning, the collector (5 mm × 50 mm) was covered with an aluminum foil and rotated at 1000 rpm. The electrospinning was performed under stable conditions of RT (21°C ± 1.5°C) and relative humidity (30% ± 5%) for 30 min per sample. The samples were removed from the collector and left to dry in vacuum overnight. The sleeves were then sterilized with either ethylene oxide (EtO) gas in vacuum, for the case of the samples used in the antimicrobial assessment, or PAA, as described for PTAs, for biocompatibility and biomechanical assessment. Sleeve sterility was assessed as described for the PTAs.

### 2.3 Histological and immunohistochemical assessment

Native and decellularized PTAs were assessed histologically and immunohistochemically, in order to evaluate the effect of decellularization and sterilization on extracellular matrix (ECM) histoarchitecture and presence of collagen IV and galactose-α-1,3-galactose-β-1,4-GlcNAc-R (α-gal), respectively. PTA samples were fixed in either 10% (v/v) neutral buffered formalin (NBF; histology), or zinc fixative (9.97 mM Tris, 2.84 mM calcium acetate, 36.68 mM zinc chloride and 22.78 mM zinc acetate dihydrate in DW, pH 7.4; immunohistochemistry) at 4°C for 24 h. The samples were dehydrated, embedded in paraffin and sectioned in 5 μm-thick slices. The sections were transferred to Superfrost Plus microscope slides (Thermo Fisher; histology), or Superfrost Ultra Plus slides (Thermo Fisher; immunohistochemistry). The sections were dewaxed, rehydrated, stained, rehydrated, mounted with coverslips, using Eukit mounting medium (Fluka), and dried overnight. For histology, sections were stained using either H&E, Roti-Mount FluorCare DAPI (Roth), Masson’s Trichrome (Sigma-Aldrich), Elastica van Gieson’s (Merck Miilipore), or Alcian Blue/PAS (Thermo Fisher), according to the manufacturers’ guidelines. Immunohistochemistry was performed using the labeled polymer technique according to the EnVision Dual Link System-HRP (DAB+) kit (Dako). The primary monoclonal antibodies used were anti-collagen IV (1:100 dilution; Dako M0785) and anti-α-gal (Enzo ALX-801-090-1; clone M86; 1:10 dilution). IgG1 (1:200 dilution) and IgM (1:60 dilution) were used as isotype controls for negative collagen IV and α-gal staining, respectively, together with omission of the primary antibody (labelled polymer-HRP). Antibodies and isotypes were diluted in .1% (v/v) bovine serum albumin (BSA; VW) in tris-buffered saline (TBS; 50 mM Tris, 150 mM NaCl, pH 7.6). Stained sections were visualized with a Nikon Eclipse TE300 microscope and images were captured with a Nikon DS-Fi1C camera.

### 2.4 Electron and multiphoton laser microscopy

Native and decellularized PTAs were assessed under TEM, whereas the polymeric sleeves were assessed under SEM, according to the method described by Rudat et al. (2014). Native and decellularized PTAs treated with PAA and γ-irradiation were cut transversely and fixed in a solution containing 1.5% (v/v) paraformaldehyde, 1.5% (v/v) glutaraldehyde, and 150 mM HEPES at pH 7.35 for 30 min at RT, followed by dehydration in increasing concentrations of acetone (30%, 50%, 70%, 90% & 100%) for 10 min each time, and embedding in Epon. Sections of 50 nm were post-stained with uranyl acetate and lead citrate, and observed under a Morgagni TEM (FEI). Images were captured with a side-mounted Veleta CCD camera. The structural and morphological characteristic of the polymeric sleeves were assessed under SEM. Sleeve samples were sputtered with an ultrathin gold layer (Polaron E5000S SEM Coating System) and images were captured using a Philips SEM-505 (Philips), at 10 kV with 1,000× magnification and 50 nm spot size. PTA scaffolds (M1-PAA & M1-γ) were further assessed under multiphoton microscopy. The multiphoton imaging was performed using a CellSurgeon multiphoton microscope (MPM; LLS-ROWIAK). The MPM comprised a femtosecond mode-locked near infrared 705–980 nm Ti:Sapphire laser (Chameleon XR, Coherent) and a microscope. The samples were visualized using a 40×/1.3 NA apochromatic oil (Carl Zeiss) or a 25×/.95 W (Leica) objective lens, and images were acquired with a voxel size of .22 × .24 × 1 μm or .42 × .44 × 1 μm, respectively, and a field of view of 700 × 700 pixels, using the built-in CellSurgeon acquisition software (LLS-ROWIAK). Collagen was visualized using two-photon excitation at 880 nm that produced a second harmonic generation signal at 440 nm. Elastin in the elastic fibers was visualized using two-photon excitation at 780 nm. Emitted light was filtered with a shortpass (E680sp-2p; Chroma Technology Corp.) or a bandpass (512-25:512/25; BrightLine HC) filter. In total, 100 images were acquired per sample.

### 2.5 Biochemical assessment

Native and decellularized PTA samples (*n* = 6 in all cases), were minced and frozen to −80°C. The samples were lyophilized overnight using a vacuum concentrator (Christ RVC 2-33 CD Plus) and weighed. DNA was extracted from ≈25 mg of wet tissue using a DNeasy Blood and Tissue Kit (Qiagen). Following elution, the DNA concentration was measured using a NanoDrop spectrophotometer (Thermo Fisher) at 260 nm. Collagen quantification was carried out by measuring the hydroxyproline (HYP) content, according to Edwards and O’Brien (Edwards and O’Brien, 1980). For this purpose, ≈15 mg from each lyophilized sample was hydrolyzed in 4 mL HCl (6 N) at 121°C overnight. The samples were incubated at 96°C until evaporation was completed, before they were reconstituted in buffer solution (13.3 g citric acid, 32 g sodium acetate, 9.1 g sodium hydroxide, 3.2 mL glacial acetic acid, 8 mL 1-propanol in 400 mL of DW; pH6.0–6.5). Trans-4-hydroxy-L-proline (Sigma-Aldrich) was diluted in buffer solution to create standard solutions (30, 25, 20, 15, 10, 8, 6, 4, 2, 1.5, 1, 0.5 μg/mL). 50 μL of each of the standard and test solutions were aliquoted into wells (*n* = 3) of 96-well plates (Nunc F) and incubated with 100 μL 1.4% (w/v) chloramine T solution (.05 mol/L; Sigma-Aldrich) for 5 min under gentle agitation. 100 μL of Ehrlich’s reagent (7.5 g 4-dimethyl-amino-benzaldehyde, 30 mL propan-1-ol, 13 mL of 62% (v/v) perchloric acid, 7 mL DW) was added to each well and incubated at 60°C for 45 min. Absorbance was measured at 570 nm with a multimode microplate reader (Biotek Synergy 2). HYP content was determined by interpolation from the trans-4-hydroxy-L-proline standard curve. Total collagen content was calculated by a HYP:collagen ratio of 1:7.14 (Harding and Wesley, 1968). Denatured HYP (dHYP) quantification was carried out according to Bank et al. (Bank et al., 1997), using α-chymotrypsin to cleave denatured collagen. Specifically, ≈15 mg of each lyophilized sample was incubated with 5 mL of α-chymotrypsin solution, containing 5 mg/mL α-chymotrypsin (Sigma-Aldrich) in digestion buffer (1.21 g Tris, .15 g CaCl2 in 100 mL DW, pH7.8), at 37°C for 24 h. The samples were centrifuged for 10 min at 600 g, and 2 mL of the supernatants were added to 2 mL of HCl (6 N) and used for dHYP quantification as in the case of HYP. Sulfated glycosaminoglycan (sGAG) content was measured according to Farndale et al. (1986). Briefly, ≈15 mg from each lyophilized sample was incubated with 5 mL papain solution containing 7 U/mL papain (Sigma-Aldrich) in digestion buffer (5 mM L-cysteine, 5 mM EDTA in PBS; pH6.0-6.1) for 24 h at 60°C under agitation. Chondroitin sulphate (CS B amino acid; Sigma-Aldrich) was diluted in assay buffer (137 mL 0.1 M sodium di-hydrogen phosphate solution, 63 mL of 0.1 M di-sodium hydrogen phosphate solution; pH6.8) to create standard solutions (30, 25, 12.5, 6.25, 3.125 μg/mL). 40 μL of each standard and test solution was aliquoted in a 96-well plate in triplicates, and 250 μL of dimethyl-methylene blue (DMB) dye solution (16 mg 1,9-dimethylene-methylene blue zinc, 2 g sodium formate, 5 mL 99.5% ethanol, 2 mL formic acid, 1 L DW; pH 3.0-3.1) was added to each well and incubated for 5 min. Absorbance was measured at 525 nm with a microplate reader; the GAG content was determined by interpolation from the CS B standard curve.

### 2.6 Biocompatibility assessment

The biocompatibility of the decellularized PTAs and polymeric sleeves was assessed under contact and extract cytotoxicity assays, according to ISO 10993-5. Both assays were performed with L929 murine fibroblasts (DSMZ ACC2), cultured in RPMI 1640 medium (Merck), supplemented with 10% (v/v) fetal bovine serum (FBS; Biochrom) and 1% (v/v) penicillin/streptomycin (P/S; Lonza), at 37°C and 5% (v/v) CO2 in air. For the contact cytotoxicity, 5 mm^2^ samples (*n* = 3) were isolated from the decellularized PTAs and polymeric sleeves, fabricated with the two PCL/PEG ratios and sterilized with PAA. Each PTA and polymer sample, together with a collagen gel (negative control; Cultrex 3D Culture Matrix Rat Collagen I; R&D Systems) and a cyanoacrylate glue (positive control; UHU) sample, were placed in individual wells of a 6-well plate, seeded with 4 × 105 cells/well L929 murine fibroblasts and cultured for 24 h at 37°C and 5% (v/v) CO2. The cells were fixed with 10% (v/v) NBF and stained with Giemsa stain (Sigma-Aldrich). Cell morphology and distribution were examined with a Nikon Eclipse TE300 microscope and images were captured with a Nikon DS-Fi1C camera.

For the extract assay, extracts were prepared by finely mincing 100 mg (wet weight) samples (*n* = 6) from the decellularized PTAs and 6 cm^2^ samples from polymeric sleeves fabricated with the two PCL/PEG ratios. The minced samples were incubated in Eppendorf tubes with 1 mL RPMI 1640 medium (with 10% (v/v) FBS and 1% (v/v) P/S) at 37°C under agitation for 72 h. After extraction, the tubes were centrifuged for 15 min at 19,000 g, and the supernatants were collected. L929 murine fibroblasts were seeded in a 96-well plate at a density of 10,000 cells/well and incubated for 24 h at 37°C in 5% (v/v) CO2 in air. The medium was replaced by 100 μL of double-strength cell culture medium (RPMI 1640, with 20% FBS and 2% P/S), together with 100 μL of each extract, or 80% (v/v) dimethyl-sulfoxide (DMSO; AppliChem) in RPMI 1640 (positive control), or RPMI 1640 (negative control), in replicates of six, and incubated for 48 h. The cells were lysed and the ATP activity was measured using an ATP-Lite kit (PerkinElmer). Luminescence was measured using a multimode microplate reader and the results were expressed as relative luminescence units (RLU).

### 2.7 Biomechanical assessment

Native (*n* = 6) and decellularized PTAs were subjected to uniaxial tensile loading to failure in order to assess the effect of the two decellularization and two sterilization methods (*n* = 6 in all cases) on the scaffolds’ integrity. Non-sterilized and PAA-sterilized polymeric sleeves fabricated with the two PCL/PEG ratios (*n* = 6 in all cases) were also subjected to uniaxial loading. Native and decellularized PTAs were tested along their axial direction only, since their small diameter did not allow for sufficiently-long circumferential specimens to be reliably tested. The sleeves were tested along their axial and circumferential direction. Testing was conducting according to Korossis et al. (2002). Samples measuring 20 × 5 mm (15 × 3 mm for the circumferential sleeve samples) were isolated from the PTAs and sleeves, and their thickness was measured using a Mitutoyo thickness gauge and averaged. The samples were mounted to a bespoke holder, which allowed sample mounting under zero stress and a sample gauge length of 10 mm (6 mm for the circumferential sleeve samples), prior to mounting them to an Instron tester (5,967 Dual Column Series; Instron) with a 100 N load cell. Testing was conducted in PBS at 37°C. During testing, the samples were preloaded to .01 N and preconditioned for 60 cycles at a strain rate 10 mm/min (PTA samples only), and then loaded to failure at the same strain rate. Failure was taken at the first decrease in the recorded load during extension. The load-extension response of each sample was converted to engineering stress-engineering strain. The biomechanical parameters calculated for the PTA samples included the elastic and collagen phase slopes, transition stress and strain, failure strain and ultimate tensile strength (UTS), whereas for the sleeve samples included the primary and secondary phase slopes, failure strain and UTS.

Native (*n* = 6) and decellularized PTAs were subjected to burst pressure testing in order to assess the effect of the two decellularization and two sterilization methods (*n* = 6 in all cases) on the burst pressure characteristics of the scaffolds. In addition, PAA-sterilized polymeric sleeves, fabricated with the two PCL/PEG ratios, were fitted on the outside of equal-length PTA scaffolds, prepared with the two decellularisation and two sterilization methods (*n* = 6 in all cases), and subjected to burst pressure testing as a dual-component graft. For this testing, decellularised PTA scaffolds with an outer diameter between 4–4.5 mm (after decellularisation) were selected and fitted with the polymeric sleeves that were manufactured with an internal diameter of 5 mm. The scaffolds with and without the polymeric sleeves, measuring 15 cm in length, were clamped on one end using adjustable stainless-steel clamps, filled with saline, and then mounted on the other end to the bottom of a pressure vessel. The pressure vessel, which could accommodate one graft at a time, was connected to a pressurized air supply at the top through an analogue pneumatic regulator and a Vivitro^®^ Labs precision digital manometer at the bottom. The pressure vessel was half-filled with saline and then sealed from the atmosphere, before the manometer was zeroed and the air pressure was increased at a rate of 100 mmHg/s. Following failure of the graft, the maximum pressure achieved was recorded in mmHg.

### 2.8 Antibacterial efficacy

The antimicrobial activity of antibiotics-impregnated polymeric sleeves was assessed with VAN and GEN against three facultative anaerobic bacteria, including *Staphylococcus Aureus* (*S. Aureus*; Gram positive; 20231 DSMZ) and *Staphylococcus Epidermidis* (*S. Epidermidis*; Gram positive; 20,044 DSMZ), as common skin bacteria, and against *Escherichia Coli* (*E. Coli*; Gram negative; 1103 DSMZ), over a period of 7 days. VAN is effective against Gram positive strains, whereas GEN is mostly effective against Gram negative strains, but also against *Staphylococcus*. The bacteria strains were subcultured in sterile TSB-medium, containing 30 g/L tryptone soya broth powder (TSB; OXOID CM0 129) and 3 g/L yeast extract powder (Carl Roth) in DW (pH7.4), at 37°C for 24 h under shaking. The suspensions were then adjusted to an optical density (OD; measured at 600 nm) of approximately .20, measured by a BioPhotometer spectrophotometer (Eppendorf), before they were used to inoculate the samples. This OD corresponded to 1 × 106 colony-forming units (CFUs)/ml for *S. epidermidis*, 1 × 108 CFU/mL for *S. aureus*, and 1 × 107 CFU/mL for *E. coli*.

Samples measuring 1 cm^2^ were isolated aseptically from the EtO-sterilized polymeric sleeves and M2-PAA scaffolds, and impregnated with antibiotics by immersing them in varying concentrations of VAN and GEN in PBS (pH7.2), and for different durations, in 50 mL Falcon tubes at RT. Specifically, samples from the 150:50 (*n* = 3 for each bacterial strain) and 100:50 (*n* = 3 for each bacterial strain) polymer blend groups were treated with 5 mg/mL VAN and 5 mg/mL GEN for either 1 h or 24 h, or with 5 mg/mL VAN and 8 mg/mL GEN for 24 h, whereas samples from PAA-sterilized PTA scaffolds (*n* = 3 for each bacterial strain) were treated with 5 mg/mL VAN and 8 mg/mL GEN for 24 h. Moreover, 1 cm^2^ samples were also isolated aseptically from the EtO-sterilized 150:50A and 100:50A sleeves (*n* = 3 for each bacterial strain in both cases) and used in the assessment without any further antibiotic treatment. Three additional 1 cm^2^ samples for each bacterial strain were isolated from 150:50 sleeves and used without any antibiotic treatment as a positive control for bacteria growth. The samples were transferred aseptically in individual wells of 6-well plates and inoculated with 4 mL of either the *S. Aureus*, *S. Epidermidis* or *E. Coli* suspensions, and incubated at 37°C and 5% (v/v) CO2 for 7 days. Wells with 1 cm^2^ samples isolated from 150:50 polymeric sleeves (*n* = 3), and incubated with 4 mL of TSB-medium only, without bacteria, were used as negative controls, whereas wells with 4 mL of TSB-medium only (*n* = 3) were used to assess medium sterility. After 24 h, 500 μL of bacterial suspension was sampled from each well and placed in standard rectangular cuvettes for measuring the OD of the bacterial suspensions. Each well was then supplemented with 500 μL of fresh TSB-medium. The sampling and OD measuring procedure was repeated every 24 h for 7 days. The daily recorded OD of each sample and bacteria strain was averaged over the number of samples in each group and plotted against time.

Moreover, a disk diffusion assay was performed in order to assess the diffusion of the antibiotics, released by the antibiotics-impregnated sleeves, through solid agar to inhibit bacterial growth. Samples measuring 1 cm^2^ were isolated aseptically from EtO-sterilized 150:50 and 100:50 sleeves, and M2-PAA scaffolds (*n* = 3 for each bacterial strain in all cases), which were impregnated with antibiotics by immersion in an solution, containing 5 mg/mL VAN and 8 mg/mL GEN in PBS (7.2), for 24 h in 50 mL Falcon tubes at RT. The choice of the concentrations of the antibiotics and treatment duration was based on the favorable results observed in the bacteria growth testing described above. Following the antibiotic treatment, the samples were placed onto bacteria lawns grown onto Muller Hinton agar (MHA) plates, which had been inoculated with either *S. Aureus*, *S. Epidermidis* or *E. Coli*. 150:50 and 100:50 sleeve and scaffold samples without antibiotics were used as negative controls of bacterial inhibition (*n* = 3 for each case and bacterial strain). The agar plates were incubated at 37°C and 5% (v/v) CO2 for 24 h, and then photographed. The bacteria inhibition area formed by each sample was calculated using ImageJ (v1.51j8; Wayne Rasband National Instituted of Health), and the results were averaged over the number of samples in each group and for each bacterial strain.

### 2.9 Data analysis

The data was analyzed in Microsoft Excel and GraphPad Prism [v6.0 (GraphPad Software Inc.)]. The results were expressed as means ± 95% confidence intervals (CI). One-way analysis of variance (ANOVA), followed by the Tukey test, was used to determine statistically significant differences between the means at the .05 level.

## 3 Results

### 3.1 Histological and immunohistochemical assessment

**FIGURE 1:**
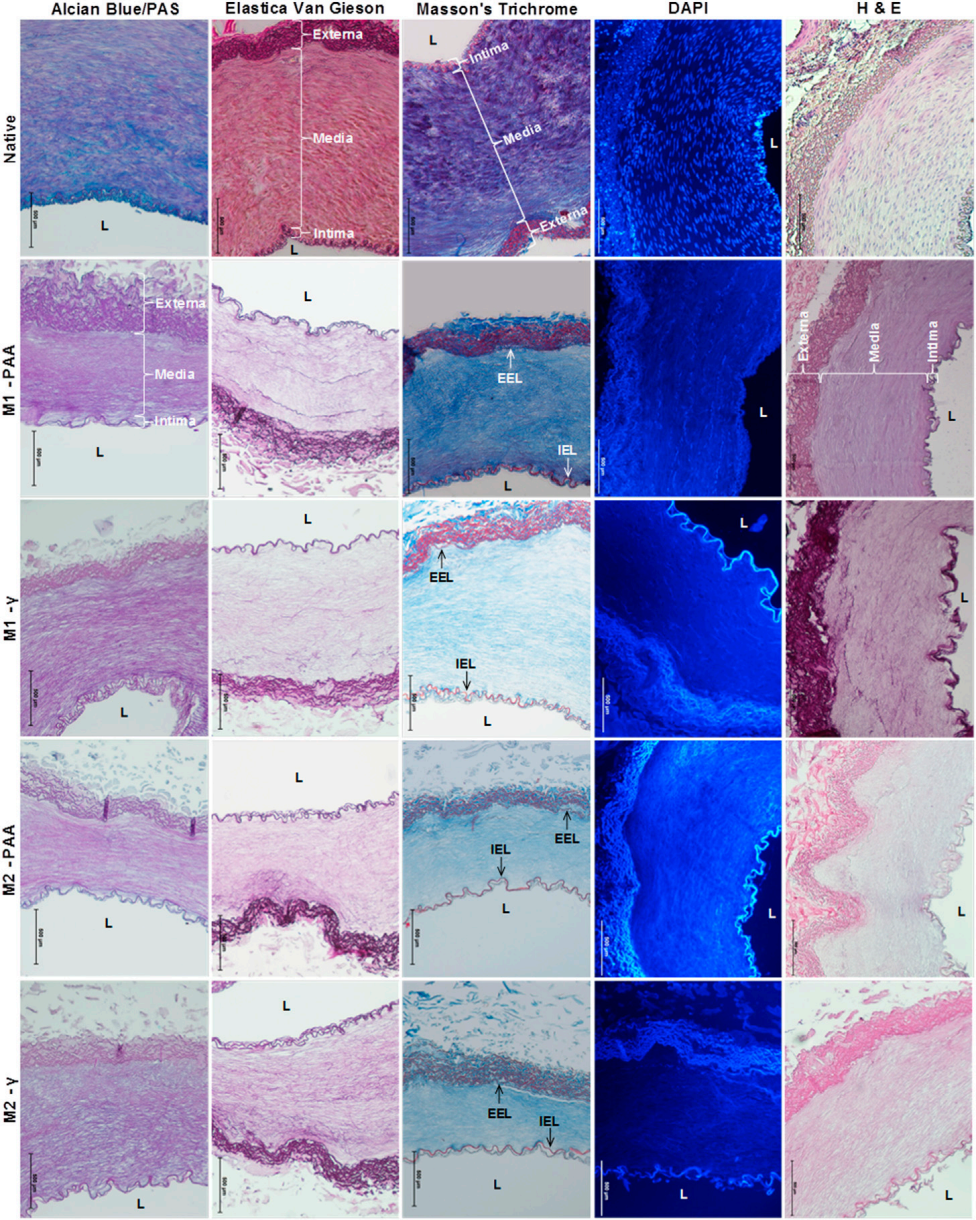
Alcian Blue/PAS, Elastica Van Gieson, Masson’s Trichrome, DAPI and H&E staining of native PTA and decellularized PTA scaffolds treated with (M2) or without (M1) RNase/DNase and sterilized with either PAA or γ–irradiation. Alcian Blue/PAS stained nuclei dark blue, acid mucosubstances (GAGs) and proteoglycans blue, and neutral polysaccharides magenta; Elastica Van Gieson stained nuclei black/brown, cytoplasm yellow/pink, elastic fibers purple and collagen light purple; Masson’s Trichrome stained nuclei dark blue/black, cytoplasm bright red, elastic fibers red and collagen fibers blue; DAPI stained nuclei blue and fluorescent under UV light; H&E stained nuclei blue/beep purple, cytoplasm red and collagen pale pink. Media: tunicae media; Intima: tunicae interna; Externa: tunicae externa; L: lumen; IEL: internal elastic lamina; EEL: external elastic lamina. All decellularized scaffolds appeared to be void of cell and cellular debris and preserved the trilaminar structure of the native tissue. It also appeared to be depleted of acid mucosubstances whilst its ECM fibers conserved their sugar moieties (Alcian Blue/PAS). The decellularized tissue also presented a well-preserved network of collagen and elastic fibers, as well as IEL and EEL. Scale bars indicate 500 μm.

**FIGURE 2:**
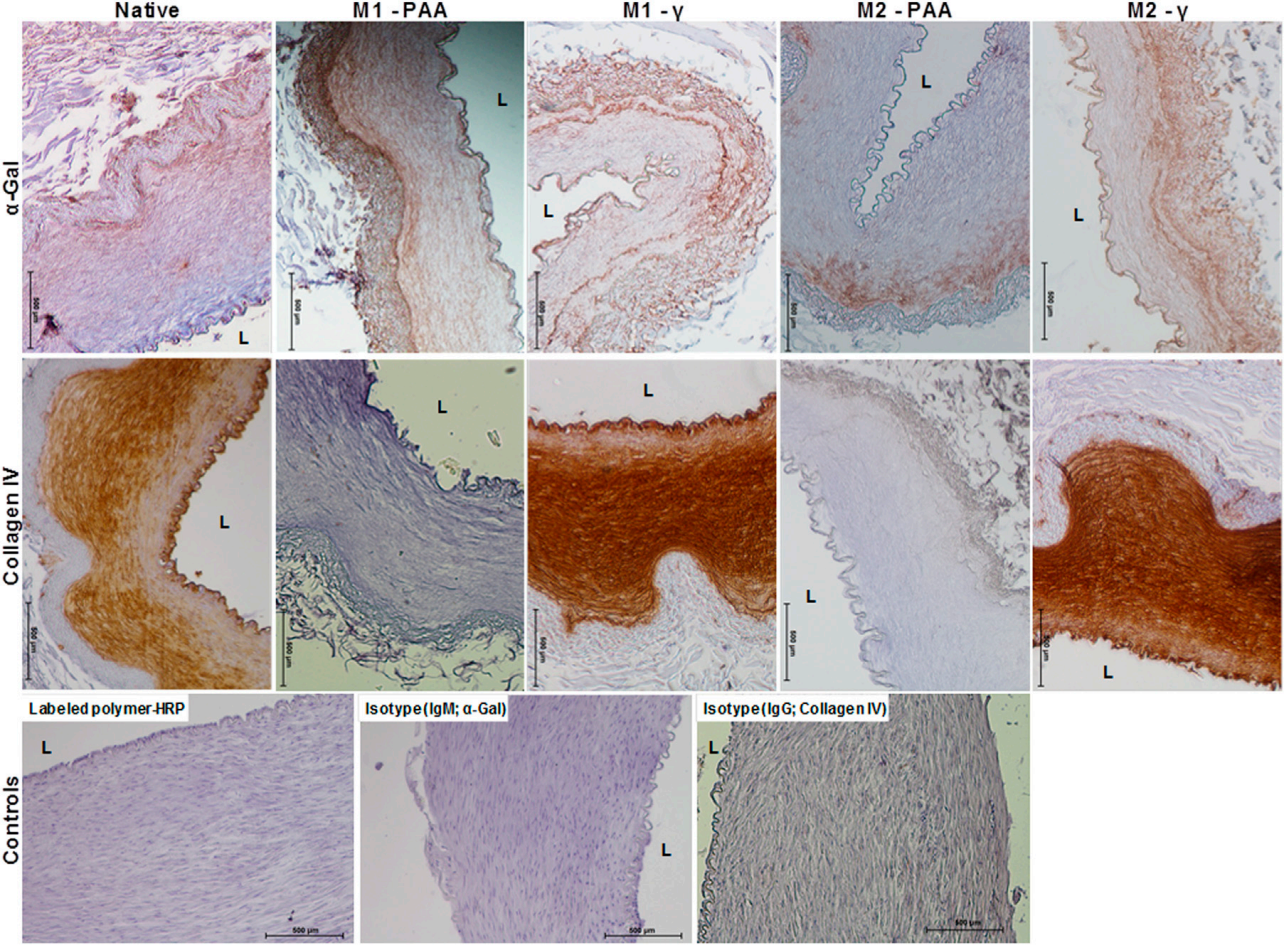
Immunohistochemical staining against α-gal and collagen IV of native PTA and decellularized PTA scaffolds treated with (M2) or without (M1) RNase/DNase and sterilized with either PAA or γ-irradiation, also showing the HRP and isotype controls. Tissue positive against the α-gal epitope was stained light brown, whereas tissue positive to collagen IV was stained dark brown. All decellularized scaffolds expressed α-gal, albeit in a reduced amount compared to the native control. Collagen IV was expressed throughout the media, intima and basement membrane of the native PTA and the decellularized PTA scaffolds that were sterilized with γ-irradiation. No staining against collagen IV could be observed in the decellularized scaffolds that were sterilized with PAA. Scale bars indicate 500 μm.

The histological and immunohistochemical results of the native and decellularized PTAs are shown in Figures 1, 2, respectively. As evidenced by all stains used, all decellularized scaffolds appeared to be void of cells and cellular debris, throughout their thickness, and preserved the trilaminar structure of the native tissue. The DAPI staining also verified the absence of cell nuclei in the scaffolds. The Alcian Blue/PAS staining indicated that the scaffolds were depleted of acid mucosubstances (GAGs) and proteoglycans, but not of neutral polysaccharides. The decellularized tissue also presented a generally preserved network of collagen and elastic fibers, albeit seemingly disrupted due the voids generated by cell removal. The scaffolds also demonstrated a preserved internal (IEL) and external (EEL) elastic laminae. The immunohistochemical staining demonstrated high presence of the α-gal xenoepitope in the scaffolds, although it was reduced compared to the native tissue. The M2-PAA scaffolds presented the least α-gal staining, with a prevalent signal only in the media. Immunohistochemical staining for collagen IV demonstrated a clearly defined collagen IV layer in the basal lamina of the luminal surface and medial layer of the native PTA. Collagen IV was strongly present in the basal lamina and media of the γ-irradiated scaffolds, but not in the PAA-sterilized ones.

### 3.2 Electron and multiphoton laser microscopy

**FIGURE 3:**
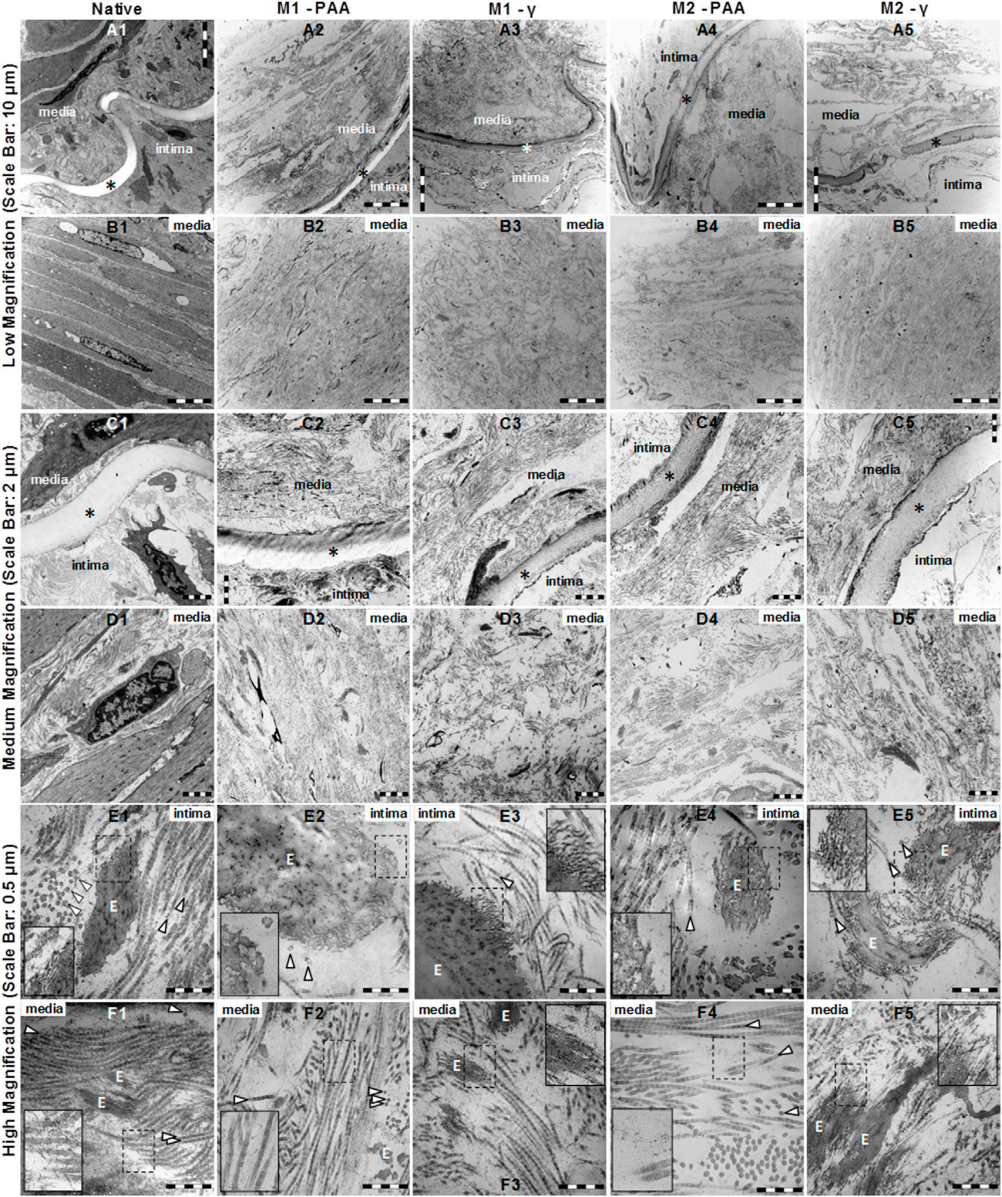
TEM imaging at low **(A1–B5)**, medium **(C1–D5)** and high **(E1–F5)** magnification of native PTA **(A1, B1, C1, D1, E1, F1)** and decellularized PTA scaffolds treated with **(M2; A4, B4, C4, D4, E4, F4, and A5, B5, C5, D5, E5, F5)** or without **(M1; A2, B2, C2, D2, E2, F2, and A3, B3, C3, D3, E3, F3)** RNase/DNase and sterilized with either PAA **(A2, B2, C2, D2, E2, F2, and A4, B4, C4, D4, E4, F4)** or γ–irradiation **(A3, B3, C3, D3, E3, F3, and A5, B5, C5, D5, E5, F5)**. Media: tunicae media; Intima: tunicae interna; È: elastic fibers; ▷ collagen fibers; ✶ interna elastic lamina. All scaffolds appeared to be completely decellularized, containing collagen and elastic fibers. Although no differences could be observed between M1 and M2, the elastic fibers of the PAA-treated scaffolds (E2, E4 nested) appeared to be cleaved of the thin fibrils that decorated the native tissue (E1 nested) and the γ-irradiated scaffolds (E3, E5 nested). In all samples, tropocollagen and collagen fibrils were evident between and around the collagen fibers (F1, F2, F3, F4, F5 nested), which were rather reduced in the case of the PAA-treated scaffolds (F2, F4, nested).

The TEM results at low, medium and high magnification of the cross-sectioned native and decellularized PTAs are shown in Figure 3. The native PTA (Figures 3A1–3E1) revealed a clearly distinguishable intima and media, containing well-organized and aligned elastic and collagen fibers, and a clearly visible internal elastic lamina (IEL) and healthy-looking cells, dispersed within a continuous collagen network. No intact cells could be identified in any of the decellularized scaffolds, whereas the organization of their collagen and elastic fibers networks, together with their IEL, seemed to be unaffected compared to the native tissue. At higher magnification, the typical cross-striation of longitudinally cut collagen fibrils could be observed throughout the decellularized scaffold, which did not differ in their ultrastructural appearance from the native tissue (Figures 3E1–3E5). In general, decellularization with (M2) or without (M1) nucleases did not generate any differences in the ultrastructural properties of the scaffolds. Both Methods resulted in completely decellularized scaffolds, with grossly preserved collagen and elastic fiber networks. However, in the PAA-treated scaffolds, the surface of the elastic fibers seemed to be different compared to the native and γ-irradiated samples. While in the native tissue and γ-irradiated samples the surface of elastic fibers was decorated with thin fibrils (Figures 3E1, E3, E5 nested), these were generally absent in the PAA-treated scaffolds (Figures 3E2, E4, nested), indicating a degradation effect by PAA. In addition, thin structures, probably representing tropocollagenssssssss and collagen fibrils, were evident between and around the collagen fibers (Figures 3F1–3F5 nested). These structures appeared clearly diminished in the PAA-treated scaffolds (Figures 3F2, F4, nested).

**FIGURE 4:**
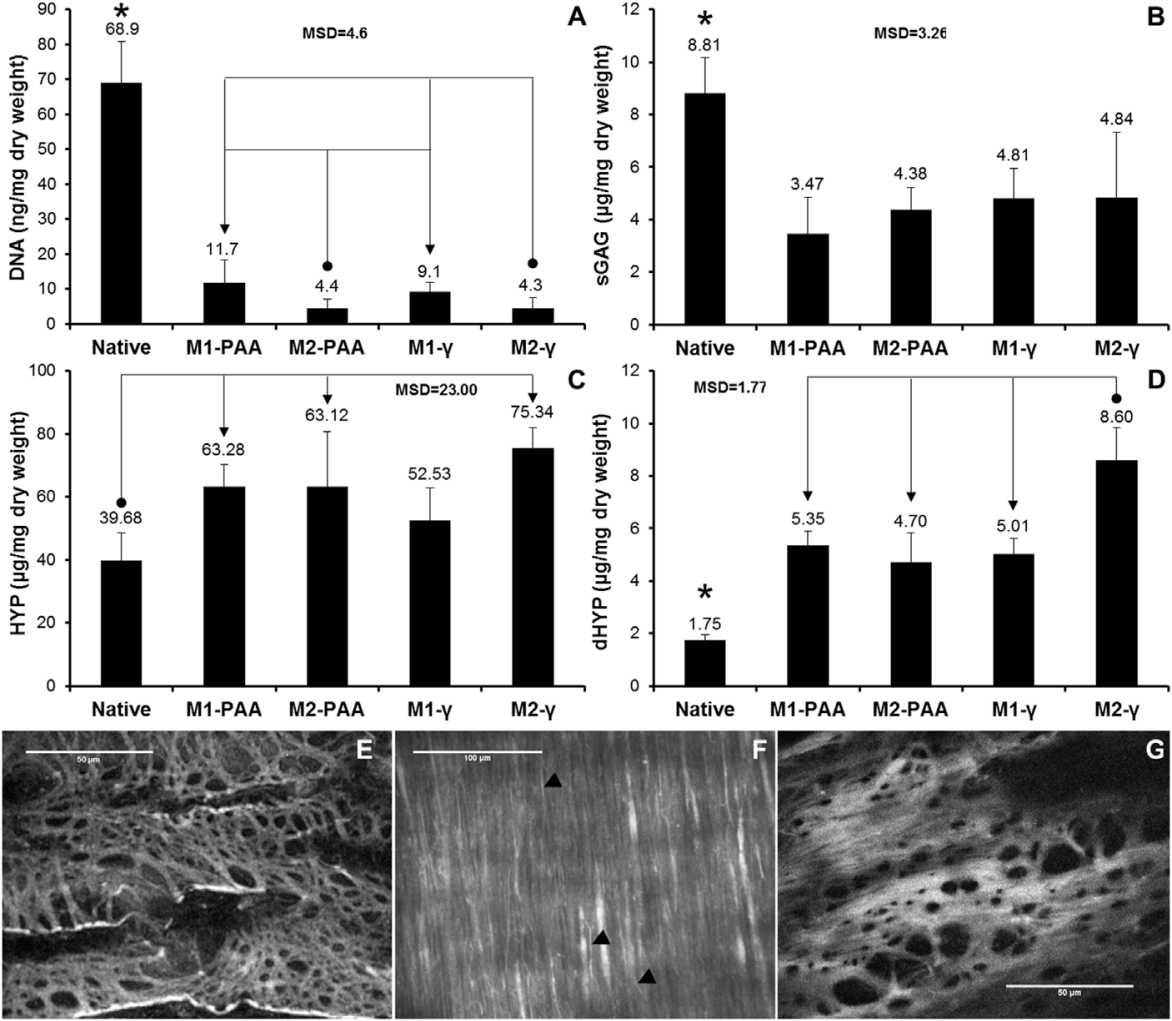
Mean DNA **(A)**, sGAG **(B)**, HYP **(C)** and dHYP **(D)** content per dry weight of native PTA and decellularized PTA scaffolds treated with (M2) or without (M1) RNase/DNase and sterilized with either PAA or γ-irradiation. Data was expressed as means (*n* = 6); error bars indicate 95% C.I.; MSD: minimum significant difference; indicates significant difference between the native and the decellularized groups. Connectors indicate significant difference between the originator and end arrow group. **(E–G)** Characteristic *en face* multiphoton images of the lumen of decellularized PTAs, treated with M1 and sterilized with either PAA **(E,F)** or γ-irradiation **(G)**, showing the elastic fiber network of internal elastic lamina (E, G; imaging plane was approximately 20 μm below lumen surface) and the collagen fibers of tunica media (F; imaging plane was about 60 μm below lumen surface). Black arrow heads (►) show elastic fibers. Scale bars indicate 100 μm **(E,F)** or 50 μm **(G)**.

The laser microscopy results included *en face* micrographs of the IEL (imaging plane ≈20 μm below lumen surface) and media (imaging plane ≈60 μm below lumen surface) of the M1-PAA and M1-γ scaffolds (Figure 4). The multiphoton images demonstrated a clearly disrupted IEL in the PAA-treated scaffolds (Figure 4E), with a shredded-like morphology, whereas the γ-irradiated scaffolds demonstrated a less-disrupted and more compact IEL (Figure 4G), with a sheet-like morphology. However, the fibrillar collagen histoarchitecture of the media did not present any significant differences between the two sterilization treatments (Figure 4G). The sleeves were assessed under SEM, to assess the effect of the two PCL/PEG mass ratios (2:1 and 3:1) on sleeve ultrastructure. These results are shown in Figure 7, for the non-sterilized 100:50 (Figure 7E) and 150:50 (Figure 7F) sleeves. The 150:50 sleeves, demonstrated larger pores and thicker more uniform fibers compared to the 100:50 sleeves, which showed variable-thickness and thinner fibers.

### 3.3 Biochemical assessment

The DNA, sGAG, HYP and dHYP contents of the native and decellularized PTAs are illustrated in Figures 4A–D, respectively. The DNA content of the scaffolds treated both with (M2) and without (M1) nucleases was significantly reduced compared to the native tissue (*p* = .000; MSD = 4.6). However, the reduction in the detectable DNA content was greater in the scaffolds treated with nucleases (93.6% and 93.7% for M2-PAA and M2-γ, respectively) than in the scaffolds treated without nucleases (83.0% and 86.7% for M1-PAA and M1-γ, respectively). The difference between the M1 groups and their corresponding M2 counterparts was statistically significant.

No statistically significant difference was observed between the two M1 or the two M2 groups. A reduction was also observed in sGAGs, with all scaffolds demonstrating significantly reduced contents compared to the native control (*p* = .000; MSD = 3.26), in accordance with the Alcian Blue/PAS staining results. There were no significant differences in the sGAG content between the scaffolds decellularized and sterilized with the different methods. In contrast, the HYP content of all scaffolds was increased compared to the native control. The increase was statistically significant for all scaffold groups, except the M1-γ group (*p* = .002; MSD = 23.00). A statistically significant increase was also observed in the case of the dHYP, with all scaffold groups demonstrating higher contents compared to the native control (*p* = .000; MSD = 1.77). In addition, the dHYP content of the M2-γ group was significantly increased compared to the other decellularized scaffold groups.

### 3.4 Biocompatibility assessment

**FIGURE 5:**
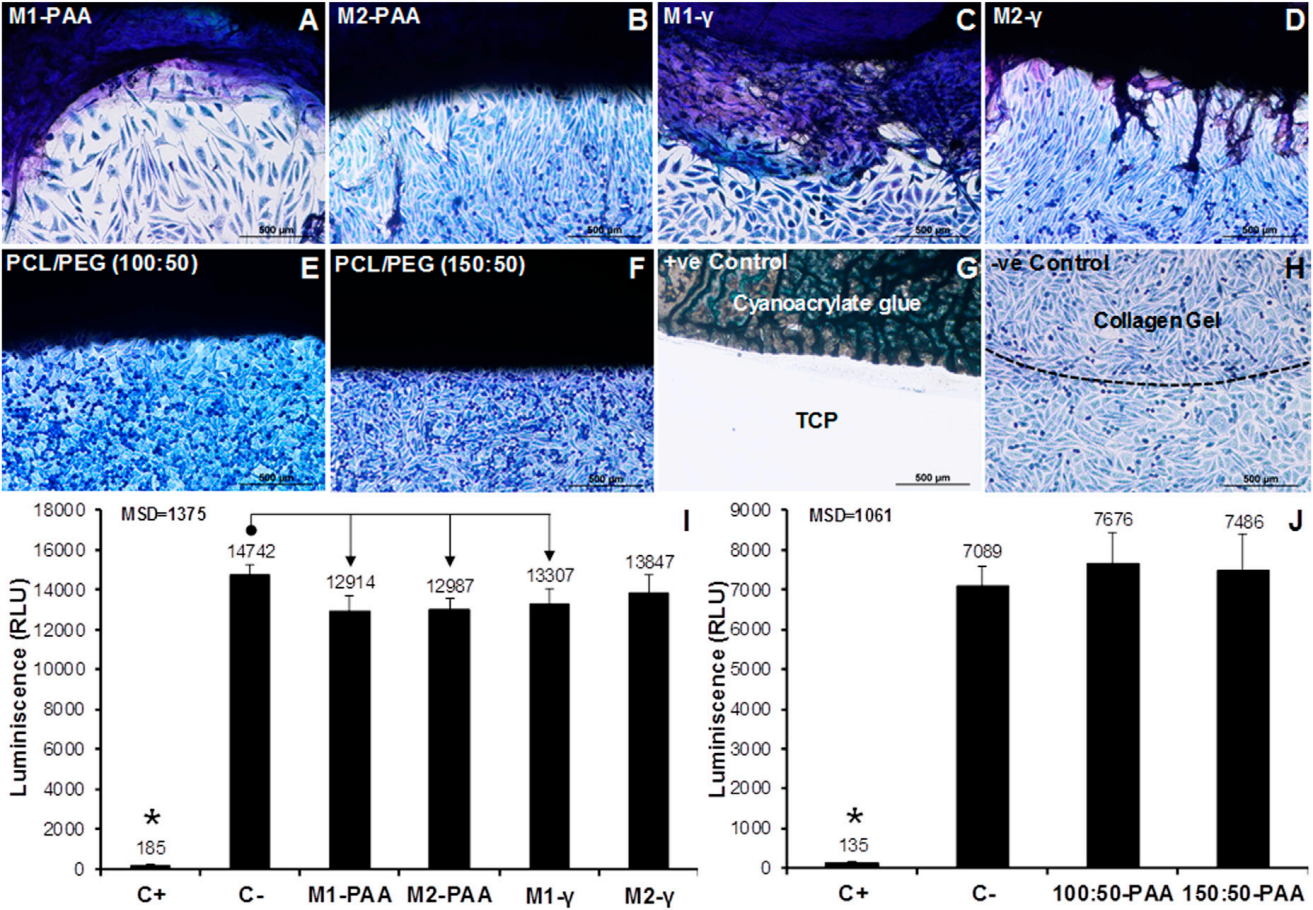
Contact **(A–H)** and extract **(I,J)** cytotoxicity results of the decellularized PTA scaffolds treated with (M2; **(B,D,I)** or without (M1; **(A,C,I)** RNase/DNase and sterilized with either PAA **(A,B,I)** or γ-irradiation **(C,D,I)**, together with the corresponding results for the 150:50 **(E,J)** and 100:50 **(F,J)** electrospun polymeric sleeves sterilized with PAA. The contact cytotoxicity **(A–H)** was performed with L929 murine fibroblasts, which were stained with Giemsa. Cyanoacrylate glue and collagen gel served as the positive (+ve) and negative (-ve) control, respectively. Scale bars indicate 100 μm. No contact cytotoxicity effects were observed with either the decellularized scaffolds or polymeric sleeves. The extract cytotoxicity assay **(I,J)** was also performed with L929 murine fibroblasts with 80% (v/v) DMSO and RPMI 1640 medium serving as positive (C+) and negative (C−) controls, respectively. Luminescence was expressed as means (*n* = 6); error bars indicate 95% C.I.; MSD: minimum significant difference; indicates significant difference between the native and decellularized groups. Connectors indicate significant difference between originator and end arrow group.

The contact cytotoxicity results of the scaffolds (Figures 5A–D) and 100:50 (Figure 5E) and 150:50 (Figure 5F) sleeves indicated that there were no cytotoxic effects on the seeded cells, which appeared confluent, growing up to and in contact with the tested materials. Similar results were observed for the case of the negative control (collagen gel; Figure 5H), but no cells survived in contact with the positive control (cyanoacrylate glue; Figure 5G). The ATP quantification indicated that both the scaffold (Figure 5I) and polymer sleeve samples (Figure 5J) achieved significantly higher ATP levels compared to the positive control (80% (v/v) DMSO) (*p* = .000, MSD = 1,375 and *p* = .000, MSD = 1,061, for the scaffolds and polymers, respectively). There was no statistically significant difference between the test and negative control (RPMI 1640) for the polymer groups. Significant differences were found between the negative control and the M1-PAA, M2-PAA and M1-γ scaffold groups, which were reduced compared to the former.

### 3.5 Biomechanical assessment

**FIGURE 6:**
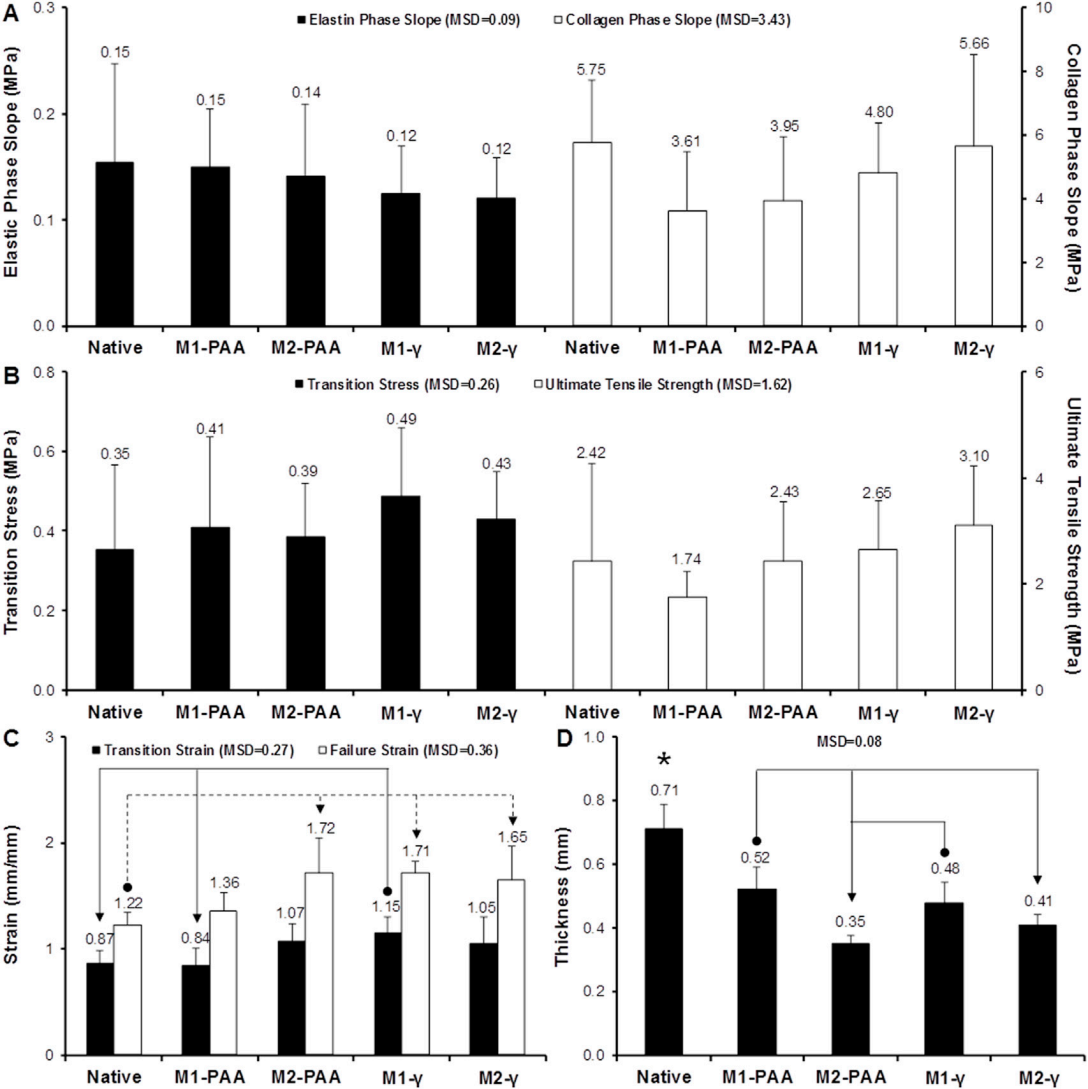
Biomechanics of native PTA and decellularized PTA scaffolds treated with (M2) or without (M1) RNase/DNase and sterilized with either PAA or γ-irradiation, tested axially. **(A)** Elastic and collagen phase slope; **(B)** transition stress and ultimate tensile strength; **(C)** transition and failure strain; **(D)** sample thickness. Data shows means (*n* = 6); error bars indicate 95% C.I.; MSD: minimum significant difference; indicates significant difference between the native and the decellularized groups. Connectors indicate significant difference between originator and end arrow group.

**FIGURE 7:**
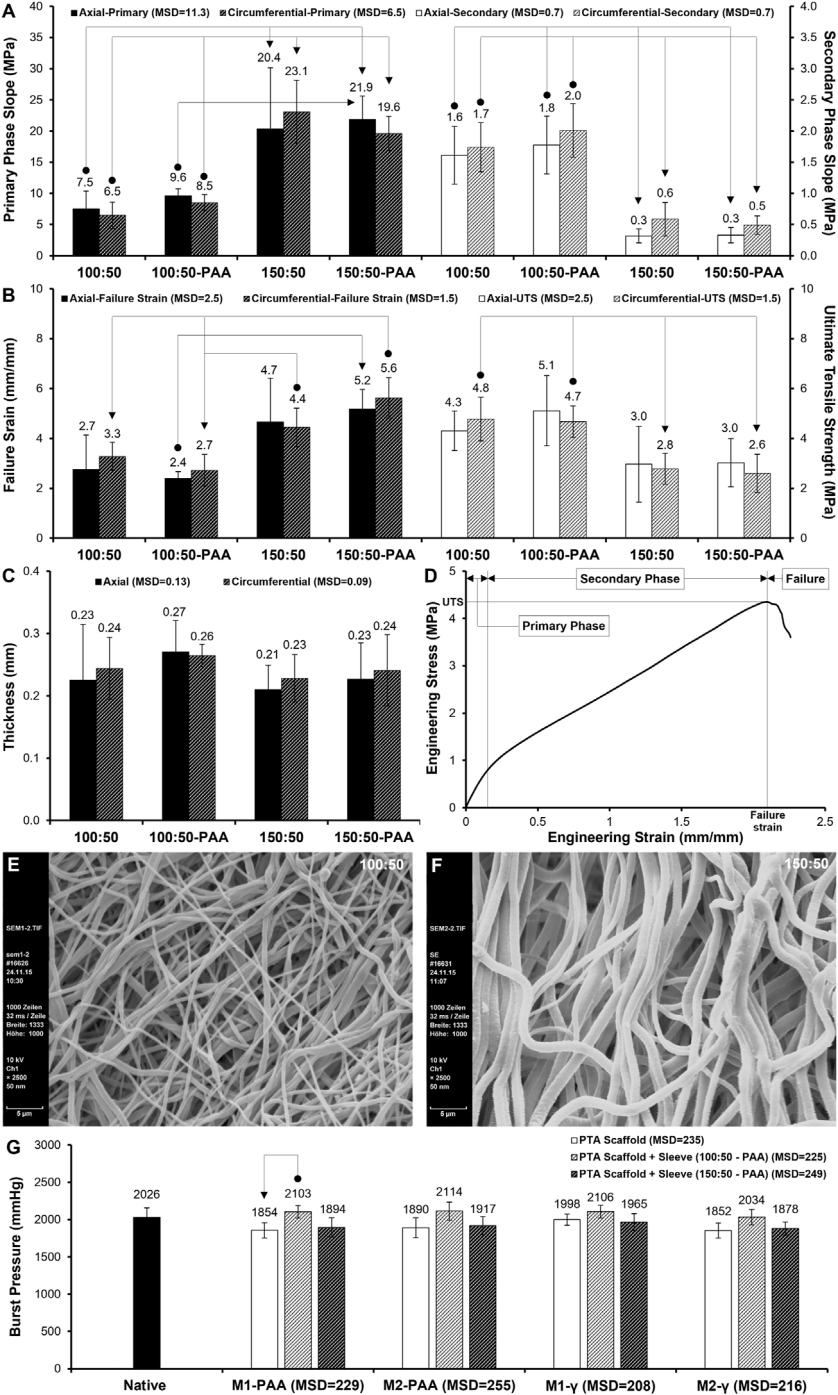
**(A–C)** Biomechanics of non-sterilized and PAA-sterilized 100:50 and 150:50 polymeric sleeves, tested axially and circumferentially. **(A)** Primary and secondary phase slope; **(B)** failure strain and ultimate tensile strength (UTS); **(C)** sample thickness. Data shows means (*n* = 6); error bars indicate 95% C.I.; MSD: minimum significant difference. Connectors indicate significant difference between originator and end arrow group. **(D)** Sample stress-strain behavior of sleeve, showing primary, secondary and failure phases. **(E,F)** SEM of 100:50 **(E)** and 150:50 **(F)** sleeves (non-sterilized). Note the increased fiber thickness and pore size of 150:50 blend. Scale bars indicate 5 μm. **(G)** Burst pressure of PTA scaffolds treated with (M2) or without (M1) RNase/DNase and sterilized with either PAA or γ-irradiation, with and without PAA-sterilized 100:50 and 150:50 polymeric sleeves. Data shows means (*n* = 6); error bars indicate 95% C.I.; MSD: minimum significant difference. Connector indicates significant difference between originator and end arrow group.

The mean axial biomechanical parameters for the native and groups are presented in Figure 6, whereas the corresponding results for the sleeves are shown in Figure 7. No significant differences were found in the elastic (Figure 6A; *p* = .750; MSD = .09) or collagen (Figure 6A; *p* = .271; MSD = 3.43) phase slopes, transition stress (Figure 6B; *p* = .647; MSD = .26) or ultimate tensile strength (Figure 6B; *p* = .211; MSD = 1.62) of the scaffold groups, compared to the native control, or compared to each other. Significant differences were only observed in the transition (Figure 6C; *p* = .010; MSD = .27) and failure (Figure 6C; *p* = .001; MSD = .36) strains, and thickness (Figure 6D; *p* = .000; MSD = .08). Specifically, all scaffold groups demonstrated significant reduced thickness compared to the native control. Moreover, the M2-treated groups demonstrated significantly reduced thickness compared to the M1-PAA and M1-γ (M2-PAA only) groups. The transition strain of the M1-γ group was significantly increased compared to the native and M1-PAA groups, whereas the failure strains of the M2-PAA, M1-γ and M2-γ were significantly increased compared to the native control. The increased transition and failure strains are indicative of an increased extensibility of these scaffolds. The typical stress-strain behavior of the sleeve samples is illustrated in Figure 7D. In order to assess this type of behavior, three distinct phases were identified, including a primary, secondary and failure phase. The primary and secondary phases were characterized by their slope, whereas the failure phase by the failure strain and UTS.

The mean biomechanical parameters for the sleeve groups tested along their axial and circumferential directions are shown in Figures 7A–C. Significant differences were found in all parameters apart from thickness (Figure 7C; *p* = .585, MSD = .13 and *p* = .704, MSD = .09 for axial and circumferential groups, respectively). No significant differences were found between the circumferential and axial directions of the two sleeve groups, indicating that they were rather isotropic. PAA sterilization did not affect sleeve biomechanics, since there were no significant differences in any of the parameters between the PAA-sterilized and non-sterilized sleeve groups. Significant differences were found between the two polymer blend ratios. Specifically, both 100:50 groups (with and without PAA sterilization) demonstrated statistically significant decreased primary phase slope (Figure 7A) compared to their 150:50 counterparts, along both the axial (*p* = .003; MSD = 11.3) and circumferential (*p* = .000; MSD = 6.5) directions, indicating that in this phase the 100:50 sleeves were more compliant. This was reversed in the case of the secondary slope (Figure 7A), with the 100:50 groups demonstrating significant increased values compared to their 150:50 counterparts, along both the axial (*p* = .000; MSD = .7) and circumferential (*p* = .000; MSD = .7) directions, indicating that in this phase the 100:50 sleeves were stiffer. In terms of the failure strain (Figure 7B), both 100:50 groups demonstrated significantly decreased means compared to the 150:50 groups, along both the axial (*p* = .011; MSD = 2.5) and circumferential (*p* = .000; MSD = 1.5) directions, indicating that the 100:50 groups were less extensible than their 150:50 counterparts. The 100:50 groups also demonstrated increased UTS compared to their 150:50 counterparts. The difference was statistically significant only along the circumferential direction (*p* = .001; MSD = 1.5).

Burst pressure testing was conducted to assess the effect of the two decellularisation methods and two sterilization techniques on the integrity of the decellularised PTAs, as well as to assess the burst pressure characteristics of the dual-component graft, comprising the PTA scaffold and polymeric sleeve. These results are presented in Figure 7G. No statistically significant differences were found among the PTA scaffold groups (without the polymeric sleeve), decellularised with M1 and M2, and sterilized with PAA and γ-irradiation, or between the PTA scaffold groups and the native control (*p* = .110; MSD = 235). This was also the case for the PTA scaffolds that were ensheathed with the PAA-sterilised polymeric sleeves. Specifically, there was no statistically significant difference between the PTA scaffold groups fitted with the 100:50 polymer blend sleeve, or between those groups and the native control (*p* = .648; MSD = 225). Similarly, there was no statistically significant difference between the PTA scaffold groups fitted with the 150:50 polymer blend sleeve, or between those groups and the native control (*p* = .424; MSD = 249). Further analysis showed that there were no statistically significant differences among the PTA scaffold groups (with and without the polymeric sleeve) treated with each of the four decellularisation/sterilization protocol combinations, or between them and the native control (M1-PAA: *p* = .023, MSD = 229; M2-PAA: *p* = .084, MSD = 255; M1-γ: *p* = .297, MSD = 208; M2-γ: *p* = .052, MSD = 216; Figure 7G), except for the case of the M1-PAA groups and between the PTA scaffold group (no sleeve) and the PTA scaffold group fitted with the 100:50 polymer blend sleeve (Figure 7G), with the latter demonstrating increased burst pressure.

### 3.6 Antibacterial efficacy

**FIGURE 8:**
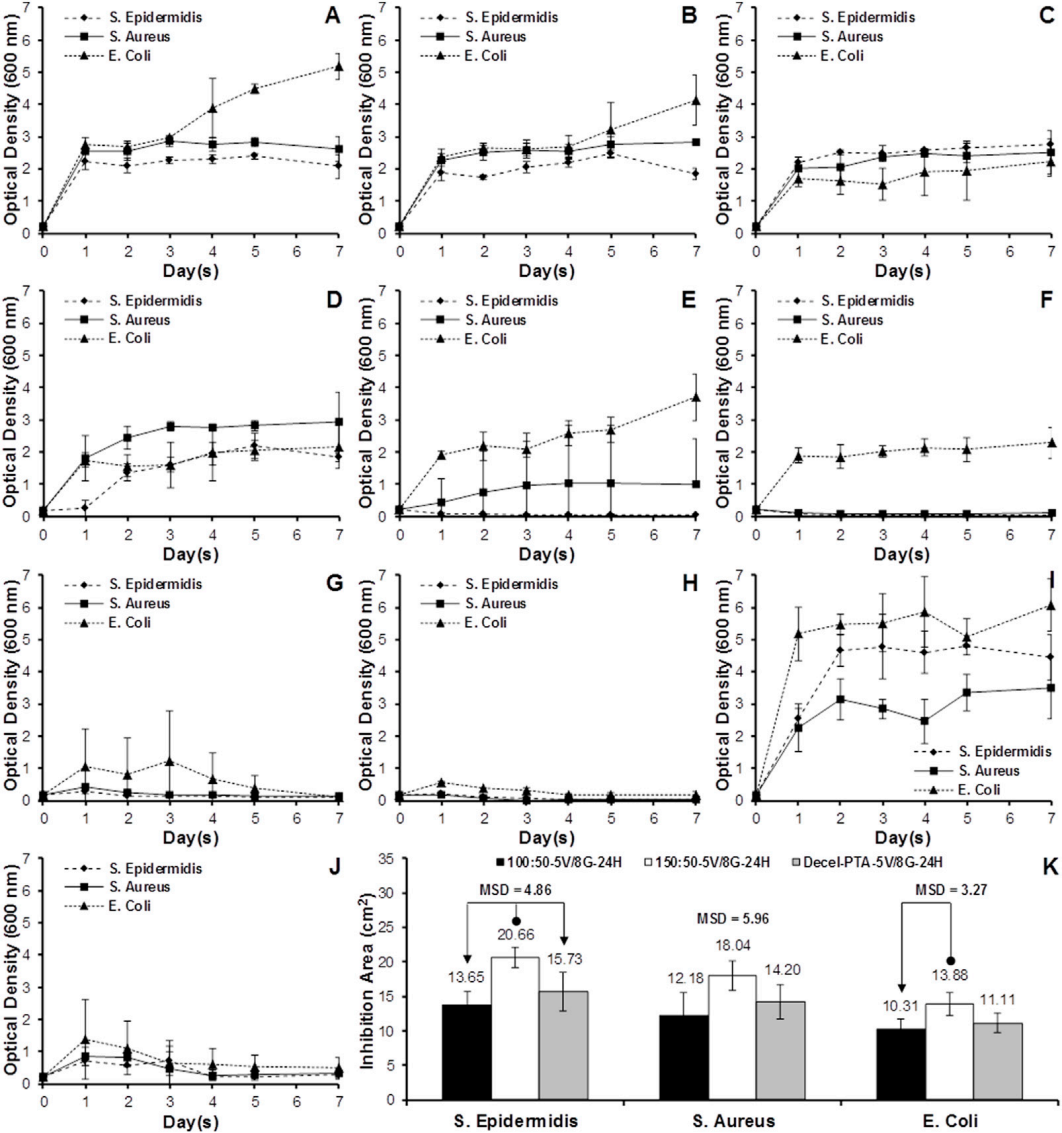
Bacteria growth curves for the polymer blends **(A–I)** and decellularized PTA **(J)**, inoculated with *S. Aureus*, *S. Epidermidis* or *E. Coli*. **(A)** 100:50A (5 mg/mL VAN and 5 mg/mL GEN supplemented in electrospinning solution); **(B)** 150:50A (5 mg/mL VAN and 5 mg/mL GEN supplemented in electrospinning solution); **(C)** 100:50-5V/5G-1H (100:50 soaked in 5 mg/mL VAN and 5 mg/mL GEN for 1 h); **(D)** 150:50-5V/5G-1H (150:50 soaked in 5 mg/mL VAN and 5 mg/mL GEN for 1 h); **(E)** 100:50-5V/5G-24H (100:50 soaked in 5 mg/mL VAN and 5 mg/mL GEN for 24 h); **(F)** 150:50-5V/5G-24H (150:50 soaked in 5 mg/mL VAN and 5 mg/mL GEN for 24 h); **(G)** 100:50-5V/8G-24H (100:50 soaked in 5 mg/mL VAN and 8 mg/mL GEN for 24 h); **(H)** 150:50-5V/8G-24H (150:50 soaked in 5 mg/mL VAN and 8 mg/mL GEN for 24 h); **(I)** 15:50-W/O (150:50 without antibiotics); **(J)** Decel-PTA-5V/8G-24H (M2-PAA decellularized PTA soaked in 5 mg/mL VAN and 8 mg/mL GEN for 24 h). **(K)** Mean bacteria inhibition area for the 100:50-5V/8G-24H, 150:50-5V/8G-24H and Decel-PTA-5V/8G-24H groups, inoculated with *S. Aureus*, *S. Epidermidis* or *E. Coli*, and cultured for 24 h in the disk diffusion assay. Data expressed as means (*n* = 3); error bars indicate 95% C.I.; MSD: minimum significant difference. Connectors indicate significant difference between originator and end arrow group.

The antibacterial activity of sleeve (100:50, 150:50, 100:50A, 150:50A) and scaffold (M2-PAA) samples, impregnated with varying concentrations of VAN and GEN for different periods, was assessed in terms of the population growth of *S. Aureus*, *S. Epidermidis* and *E. Coli* over a period of 7 days. These results are shown in Figure 8A–J, together with the corresponding results of the 150:50 polymer sleeve samples, which were not treated with any antibiotics, and used as a as a positive control (150:50-W/O; Figure 8I). Groups 100:50A (Figure 8A) and 150:50A (Figure 8B), which were fabricated with 5 mg/mL VAN and 5 mg/mL GAN supplemented in the electrospinning solution, were able to produce a certain degree of bacterial inhibition compared to the positive control (150:50-W/O), especially for the *S. Epidermis* and *E. Coli* strains. However, all strains were able to proliferate and sharply increase their OD after 24 h, reaching a plateau that was maintained after 7 days. A similar bacterial growth behavior was observed for the 100:50 and 150:50 samples, which were incubated with 5 mg/mL VAN and 5 mg/mL GEN for 1 h (100:50-5V/5G-1H, Figure 8C; 150:50-5V/5G-1H; Figure 8D). Increasing the incubation time of the 100:50 and 150:50 samples with the same concentration of VAN and GEN to 24 h (100:50-5V/5G-24H, Figure 8E; 150:50-5V/5G-24H; Figure 8F), the polymer samples were able to inhibit the growth of *S. Epidermis* and *S. Aureus*, and even completely suppress *S. Epidermis*, whereas the 150:50-5V/5G-24H group was also able to completely suppress *S. Aureus*. Neither of the 100:50-5V/5G-24H and 150:50-5V/5G-24H groups was able to effectively inhibit *E. Coli* growth, although they reduced it compared to the 150:50-W/O group.

In order to increase bacterial inhibition, the polymer samples were incubated with 5 mg/mL VAN and 8 mg/mL GEN for 24 h (100:50-5V/8G-24H, Figure 8G; 150:50-5V/8G-24H; Figure 8H). Both groups were able to completely suppress all three strains after 7 days, with the 150:50-5V/8G-24H group producing a faster suppression compared to the 100:50-5V/8G-24H. The scaffolds that were incubated with 5 mg/mL VAN and 8 mg/mL GEN for 24 h (Decel-PTA-5V/8G-24H; Figure 8J) also showed a prominent bacterial inhibition. However, complete bacterial suppression could not be achieved after 7 days. The three best performing groups (100:50-5V/8G-24H, 150:50-5V/8G-24H, Decel-PTA-5V/8G-24H) were used in the disk diffusion assay (Figure 8K). The 150:50-5V/8G-24H group demonstrated increased inhibition areas compared to the 100:50-5V/8G-24H and Decel-PTA-5V/8G-24H, for all three strains. The increase was statistically significant only for *S. Epidermis* (*p* = .011; MSD = 4.86) and *E. Coli* compared to the 100:50-5V/8G-24H group (*p* = .034; MSD = 3.27).

## 4 Discussion

To the best of the authors’ knowledge, this was the first study on the development of a dual-component infection-resistant arterial replacement for small-caliber reconstructions, comprising a decellularized porcine tibial artery scaffold and an antibiotic-releasing polymeric outer sleeve. Previously, there have been two studies by Gong et al. (2016) and Yang et al. (2019), reporting on the development of a dual-component small-caliber graft, using a similar approach to the present study. Both studies, which were conducted by the same group, utilized rodent (rat) abdominal aortas, which were decellularised using a detergent-based protocol, combined with vacuum freeze-drying. The rat abdominal aorta has little relevance in the clinical setting for the development of a small-caliber vascular graft due to its short length, whilst it cannot be used as a model of the human abdominal aorta since the latter cannot be used as a small-caliber vascular graft in the clinical setting. The present study developed and characterized a tibial artery scaffold, which was decellularized according to a modified version of the in-house protocol, with and without nucleases, and sterilized by either PAA or γ-irradiation. The present study utilized PTA as a model of the human tibial artery, which can be used clinically as a homologous small-caliber arterial replacement. In addition, decellularized PTAs could be potentially used in the clinical setting, providing that they are free of immunogenic xenoepitopes, especially α-gal. Furthermore, the decellularisation protocol that was utilized in the previous studies demonstrated significantly inferior performance in terms of DNA removal, which is a potent marker of decellularization efficacy. Specifically, the reduction of the DNA content in the decellularised scaffolds compared to the native tissue that was reported in those studies was 70% (Gong et al., 2016), whereas in the present study the reduction in the DNA content in the developed scaffolds compared to the native tissues was up to 93.7%. Both previous studies utilized polycaprolactone (PCL) fibers that were electrospun directly on the outer surface of the decellularized rat aorta. In contrast, the approach followed in the present study was to manufacture the decellularised scaffold and polymeric sleeve separately, giving significant flexibility in both the fabrication of the polymeric sleeve and the storage of the two components (decellularised scaffold and polymeric sleeve) of the dual-component arterial graft, which would most probably require different long-term storage conditions for their off-the-self availability and the successful clinical application of the dual-component graft.

Moreover, the electrospun PCL sleeve that was developed in the study by Gong et al. (2016) was used purely for the mechanical support of the decellularised scaffold, which demonstrated a significant reduction in strength compared to its native counterpart (Gong et al., 2016). In the subsequent study by the same group, the PCL nanofibers were blended with rapamycin to fabricate a dual-component graft with a drug delivery function to prevent intimal hyperplasia (Yang et al., 2019). On the other hand, the present study developed an antimicrobial mechanism for tackling the high rate of infections, associated with vascular grafting. To the best of the authors’ knowledge, the antimicrobial mechanism proposed in the present study has not been investigated before. The work developed and characterized electrospun polymer blend tubular sleeves, fabricated with two PCL/PEG mass ratios, for engaging the scaffold and providing a local antibiotic-releasing mechanism at the site of implantation. Polymeric sleaves fabricated with PCL/PEG blends have the potential to better tailor drug release *in situ* after implantation, in order to control the antimicrobial activity induced by the graft. The antibiotics were loaded onto the sleeves by either fabricating them with antibiotics supplemented in the electrospinning solution, or by soaking them in antibiotics solution after electrospinning. Moreover, the effect of PAA-sterilization on the cytotoxicity and biomechanics of the polymer sleeves was also assessed, as a simple benchtop technique that could be applied for the treatment of the dual-component graft incorporating both the decellularized scaffold and polymer sleeve, without any specialized equipment.

PAA and γ-irradiation are two commonly used sterilization methods for biological tissues (Qiu et al., 2011). PAA has been reported to be bactericidal at .001% (v/v), fungicidal at .003% (v/v) and sporicidal at .3% (v/v) (Crow, 1992). In the present study, PAA was used at a concentration of .1% (v/v), which was sufficient to sterilize the PTAs and polymer sleeves. In the case of γ-sterilization, a dose of 25 kGy has been routinely applied for medical devices and biological tissues (da Silva Aquino, 2012). However, recent studies have reported that bovine decellularized pericardium (Guhathakurta et al., 2016) and human tendon, tracheal, skin and amnion (Harrell et al., 2018) treated with 25 kGy demonstrated a significant mechanical and biological deterioration, whereas porcine aortic heart valves treated with as low as 1 kGy demonstrated significant biomechanical and ultrastructural changes *in vitro*, and significant deterioration after only 2 months *in vivo* (Helder et al., 2016). In the present study, a dosage of 150 Gy was sufficient to sterilize the decellularized PTAs, as assessed by sterility testing. However, it should be noted that following isolation, and prior to decellularization, the PTAs were disinfected by incubation with an antibiotics solution. This would have reduced the bioburden of the PTA scaffolds prior to sterilization with either PAA or γ-irradiation.

Histology confirmed that the scaffolds, regardless of whether they were treated with (M2) or without (M1) nucleases, or sterilized by PAA or γ-irradiation, were void of cells, nuclei and cellular debris, and generally preserved the trilaminar structure of the native tissue, with a seemingly well-preserved networks of collagen and elastic fibers. Some disruption in the ECM was observed, but this was due to the voids generated by the cell removal. However, the scaffolds were also depleted of acidic mucins, indicating depletion of GAGs, although neutral polysaccharides were retained in the ECM. This has also been reported by previous studies that used detergent-based decellularization (Luo et al., 2014; Granados et al., 2017). The scaffolds also demonstrated a preserved IEL and EEL elastic laminae, with the former being an important feature for endothelial cell attachment and resistance to thrombus formation (Wilshaw et al., 2012). Nevertheless, under multiphoton microscopy, and whilst the γ-irradiation scaffolds demonstrated a IEL with a sheet-like morphology, resembling that of native arteries that was reported in previous studies (Neufeld et al., 2013), the IEL of the PAA-treated scaffolds was found to be significantly disrupted, demonstrating a shredded-like morphology. However, multiphoton microscopy showed that the collagen histoarchitecture in the media was similar between the two sterilization treatments. Detrimental effects in the PAA-treated samples could also be observed under TEM. Although at low magnification all scaffolds were void of cells and cellular debris, with well-maintained collagen and elastic fiber networks, at high magnification the surface of the elastic fibers of the PAA-treated scaffolds seemed to be depleted of thin fibrils that decorated the native and γ-irradiated samples. Similar differences were observed in the collagen, with the PAA-treated scaffolds lacking thin structures, probably representing tropocollagen and collagen fibrils, which were present in the native and γ-irradiated samples.

Immunohistochemical staining showed that collagen IV was strongly present in the basal lamina, intima and media of the native, M1-γ and M2-γ samples, but not in the PAA-sterilized scaffolds, in which collagen IV staining was completely absent. This indicated that the PAA-treated scaffolds were either depleted of collagen IV, or the collagen IV structure was altered by the PAA treatment and rendered unrecognizable by the anti-collagen IV antibody. Collagen IV presence has been suggested to promote scaffold endothelialization (Brown et al., 2006). However, decellularized pulmonary valves treated with trypsin/EDTA, which was shown to remove the basement membrane, were successfully re-endothelialized *in vitro* (Cebotari et al., 2002). The immunohistochemical staining also demonstrated a high presence of the α-gal xenoepitope in the scaffolds treated with both M1 and M2, albeit reduced compared to the native tissue. The M2-PAA scaffolds presented the least α-gal staining, present only in the media of the scaffolds. Successful clinical translation of xenogeneic scaffolds necessitates the effective removal of xenoantigens, especially α-gal, which has been identified as responsible for hyperacute xenograft rejection in humans (Simon et al., 2003). It has been suggested that apart from cellular membranes, α-gal is also present on ECM proteins and proteoglycans of xenogeneic tissues. As such, complete removal of α-gal might not be possible by decellularization alone (Galili, 2015). This xenoepitope could be selectively removed by α-galactosidase, or α-gal-knockout pig tissue could be used for scaffold development. In animal tissue, however, additional xenoantigens, such as N-glycolylneuraminic acid, are present that have been linked to chronic and systemic inflammation (Samraj et al., 2015).

In addition, decellularized scaffolds should be void of DNA, since they have been linked to inflammatory responses (Keane et al., 2012), whereas large DNA fragments could potentially act as nucleation sites for calcification (Wilshaw et al., 2012). DNA reduction is an important measure of the effectiveness of a decellularization protocol, whereas absence of a substantial DNA content in a scaffold is highly desirable for clinical application (Wilshaw et al., 2012). The DNA content of both the M1-and M2-treated scaffolds was significantly reduced compared to the native tissue, with the reduction being significantly greater in the scaffolds treated with nucleases than those treated without. Gilbert and colleagues used the PicoGreen assay to quantify double-stranded DNA in commercially available biological scaffolds, and found small amounts of residual DNA in most of them. Based on this, residual DNA lengths of less than 300 bp, and DNA contents of less than 50 ng/mg of dry tissue have been considered acceptable (Gilbert et al., 2009). The residual amount of DNA obtained with the Nanodrop spectrophotometer in the present study for the M2-PAA and M2-γ scaffolds was 4.4 ± 2.6 and 4.3 ± 3.2 ng/mg, respectively (Figure 4A). Similar values of residual DNA have been reported for decellularized human common femoral arteries (7 ± 1 ng/mg) (Wilshaw et al., 2012) and human aortas (5 ± 1.5 ng/mg) (Aldridge et al., 2018). The DNA amount measured for the native PTAs was 68.9 ± 11.9 ng/mg, which corresponds relatively well to the DNA values of 273 ± 72 ng/mg and 88 ± 43.4 ng/mg, reported previously for the native human common femoral artery (Wilshaw et al., 2012) and native human aorta (Aldridge et al., 2018), respectively. It should be noted that unlike the PicoGreen assay, which is more sensitive, but limited to only detecting double-stranded DNA, the spectrophotometric method used in the present study was able to detect both single- and double-stranded DNA, giving a better quantification of the DNA content.

With regards to HYP, the mean content measured for the native PTAs was 39.68 ± 8.80 μg/mg of dry tissue. Considering that HYP constitutes 1/7.14 of collagen, the content measured corresponds to a collagen content of 283.34 ± 62.83 μg/mg of dry native tissue. This value corresponds well to the collagen contents of 173.6 ± 29.4 μg/mg and 259.18 ± 21.42 μg/mg, reported previously for the native human aorta (Aldridge et al., 2018) and native porcine pulmonary artery (Luo et al., 2014). Similar comparability was found in the mean dHYP and sGAG content of the native PTA, with the values obtained in the present study (1.75 ± .18 and 8.81 ± 1.36 μg/mg of dry tissue, respectively) corresponding well to the mean dHYP and sGAG content of native porcine pulmonary artery (2.8 ± .3 and 9.7 ± 1.2 μg/mg of dry tissue, respectively), reported by Luo et al. (2014). The mean HYP and dHYP contents of all scaffolds were significantly increased compared to the native control. No significant difference was found between the decellularized groups, except for the case of the mean dHYP content of M2-γ group, which was significantly increased compared to the other scaffold groups, indicating a possible damaging effect of the M2-γ treatment on the collagen fibers. The increase in the HYP and dHYP contents of the decellularized compared to the native PTAs was most probably due to the loss of tissue components during decellularization, which affected the overall constitution of the scaffolds. Specifically, and in addition to the removal of the cellular mass, the scaffolds were also depleted of a significant amount of their GAG content, as evidenced both by the Alcian Blue/PAS staining and the biochemical results, which showed that the mean sGAG content of all decellularized groups was significantly reduced, compared to the native control. The loss of these components increased the relative amount of HYP per unit mass of the scaffolds. The decrease in the GAG and the increase in the HYP content following decellularization, has been reported by previous studies with detergent-based decellularization (Korossis et al., 2002; Wilshaw et al., 2012; Luo et al., 2014; Granados et al., 2017). No significant differences were observed in the sGAG content between the scaffolds decellularized and sterilized with the different protocols.

Previous studies have suggested that the loss of GAGs contributes to changes in the mechanical properties of the tissues through disruption of their interaction with other ECM components (Sacks et al., 2011; Luo et al., 2014). GAGs interact with collagen and elastic fibers and cells, and form proteoglycans, which have been reported to contribute to collagen assembly and crimping, and mediate the formation of mature collagen fibers. It has been suggested that fibrillogenesis of collagen I is regulated by GAGs, which are entrapped within the collagen fiber network (Yoon and Halper, 2005). GAGs have been reported to confer increased stiffness to and attract water into the ECM, giving the tissue turgor (Sacks et al., 2011). The biomechanical analysis in the present study indicated a trend of reduced elastic and collagen phase slope (indicating increased scaffold extensibility) for the γ-irradiated and PAA-treated scaffold groups, respectively. However, these decreases were not statistically significant. Nevertheless, it should be noted that the thickness of the decellularized PTA groups was significantly decreased compared to the native control, most probably due to the GAG loss, which made the samples more compressible by the thickness measuring device. Taking this into account and calculating the elastic and collagen phase slopes of the force-strain plots (instead of the stress-strain plots) of the native and decellularized PTA samples, in terms of the force generated per unit strain (N/mm/mm), statistically significant differences were found between the native and decellularized PTA groups. Specifically, the mean values calculated for the elastic phase slope of the native, M1-PAA, M2-PAA, M1-γ and M2-γ groups were .55 ± .24N, .38 ± .12N, .24 ± .11N, .29 ± .10N, and .25 ± .08N, respectively, whereas the corresponding values for the collagen phase slope were 20.58 ± 8.07N, 9.19 ± 4.07N, 6.94 ± 3.77N, 11.29 ± 3.30N and 11.86 ± 7.08N, respectively. With regards to the elastic phase slope, the mean values of the M2-PAA, M1-γ and M2-γ groups were significantly decreased compared to the native control (*p* = .002; MSD = .23), whereas in the case of the collagen phase slope, only the PAA-treated groups (M1-PAA and M2-PAA) demonstrated significantly decreased values compared to the native control (*p* = .002; MSD = 9.35). Similar significant differences were also found in the case of the transition and failure strains (not a function of the thickness of the sample, but only of its length). These findings indicated that the decellularized groups were more extensible compared to the native control in both the elastic and collagen phases. The increased extensibility of the scaffold groups in the elastic phase could be potentially attributed to the GAG and proteoglycan loss, which allowed the ECM to deform more freely. The GAG and proteoglycan loss could also have caused the increased extensibility in the collagen phase of the PAA-treated samples, which they also showed the highest GAG reduction, together with collagen IV loss. No significant differences or trends were found between the decellularized groups and the native control in the transition stress and ultimate tensile strength. The latter was also reflected in the burst pressures of the PTA scaffolds. Specifically, none of the decellularisation/sterilization protocol combinations produced and significant changes in scaffolds compared to the native PTA control (Figure 7G).

The contact cytotoxicity assessment of the decellularized PTA scaffolds and the polymer sleeves indicated that there were no cytotoxic effects on the seeded L929 fibroblasts. However, in the case of the extract cytotoxicity with the PTA scaffolds, and even though their ATP levels was significantly higher than the positive control [80% (v/v) DMSO], a cytotoxic effect was observed in the case of the M1-PAA, M2-PAA, and M1-γ groups, whose ATP levels were significantly reduced compared to the negative control (RPMI 1640). This could be potentially indicative of residual detergents in the scaffolds, and the need for increased washing cycles for effectively removing detergent residues. No cytotoxic effects were observed in the extract cytotoxicity assessment with the sleeve samples. Moreover, under SEM the 150:50 sleeves, demonstrated a more uniform structure with thicker fibers and larger pores, compared to the 100:50 sleeves, which showed a variable fiber thickness and generally thinner fibers. These differences could potentially have an effect on the degradation rates of the polymer sleeves. The increased porosity of the 150:50 sleeves could have potentially increased the intake of antibiotic solution during soaking, which, in turn, caused a higher release of antibiotics. In addition, the increased fiber thickness of the 150:50 blend indicated an increase in the specific area of the fibers, which would have increased the amount of antibiotics that physically bound to the polymer during soaking. These features of the 150:50 sleeves could have potentially contributed to their superior antimicrobial performance.

The differences found in the structure of the sleeves fabricated with the two polymer blends were manifested in their mechanical properties. Both polymer blends demonstrated biphasic stress-strain behaviors, with a primary high-modulus linear phase and a secondary low-modulus linear region. The 150:50 group demonstrated significantly higher primary phase slope and failure strain, and significantly reduced secondary phase slope and ultimate tensile strength, compared to the 100:50 group, demonstrating the “tailorability” of the biomechanics of the sleeve using different PCL/PEG mass ratios. The biomechanical assessment did not demonstrate significant differences between the axial and circumferential directions of the two polymer blends, indicating that they were quite isotropic. Moreover, the PAA treatment did not affect the biomechanics of the polymer groups, whereas there were no differences in the thickness between any of the groups tested, indicating the high repeatability of the electrospinning technique. Comparing the physical properties of the two components, the thickness of the sleeves was about half the thickness of the PTA scaffolds. This can be considered an advantage, given the limited space at the implantation site and the minimal disturbance that needs to be imposed on the surrounding tissues. However, there was a significant modulus mismatch between the scaffolds and the sleeves along their axial direction, especially in the case of the 150:50 sleeves, with the sleeves demonstrating higher slopes (less compliant) at low strains (primary phase) than the scaffolds at low strains (elastic phase). On the other hand, the axial collagen phase slope (high strains) of the scaffolds was higher than the axial secondary phase slope of the sleeves, indicating that at higher strains the sleeves would impose little resistance to scaffold dilation under the *in vivo* pulsatility. The scaffolds and the 100:50 sleeves demonstrated comparable axial strains to failure, whereas both the 100:50 and 150:50 sleeves demonstrated higher axial ultimate tensile strengths compared to the PTA scaffolds.

Comparison of the biomechanical behavior of the scaffolds and polymer sleeves along their circumferential direction is more important for understanding their functional mismatch/compatibility under pulsatile flow and subsequent graft dilation *in vivo*. In the present study, only the axial direction of the scaffolds was tested, since it was not possible to assess the biomechanics of the PTAs along their circumferential direction, due to their small diameter. Nevertheless, previous studies have shown that the circumferential direction of the blood vessels, which is the main alignment direction of the collagen fibers in the media, is considerably stiffer than the axial direction. Korossis et al. (2005) reported mean elastic phase slopes of .16MPa and .29 MPa for the axial and circumferential direction, respectively, and mean collagen phase slopes of 1.26 MPa and 3.78 MPa for the axial and circumferential direction, respectively, for the native porcine aorta. Luo et al. (2014) reported mean elastic phase slopes of .03 MPa and .04 MPa for the axial and circumferential direction, respectively, and mean collagen phase slopes of .51 MPa and .79 MPa for the axial and circumferential direction, respectively, for the native porcine pulmonary artery. Therefore, it can be safely concluded that the circumferential direction of both the native and decellularized PTAs would demonstrate higher elastic and collagen phase slopes compared to their axial direction. This would reduce the modulus mismatch between the scaffolds and sleeves at low strains. Moreover, the decellularized PTAs in the present study demonstrated increased extensibility compared to their native state. This has also been reported by several previous studies, for both the axial and circumferential direction of blood vessels decellularized with detergents (Korossis et al., 2005; Luo et al., 2014). Considering this, and although a reduced distensibility of the sleeve might be regarded problematic in restricting scaffold dilation *in vivo*, a modulus mismatch between the sleeve and the scaffold might actually be beneficial in preventing potential aneurysmatic behavior of the scaffold. In any case, and since the predominant function of the polymer sleeve would be to induce antimicrobial activity *in situ*, a setup could be considered in which the polymer sleeve is sized so that its internal diameter is larger than the outer diameter of the PTA scaffold, so that the former does not obstruct the dilation of the latter. Alternatively, the sleeve could be incised axially, so that to minimize the circumferential resistance imposed on scaffold dilation. In terms of the burst pressure characteristics of the dual-component graft, comprising the PTA scaffold and polymeric sleeve, none of the four scaffold decellularisation/sterilization combinations (M1-PAA, M2-PAA, M1-γ, M12-γ), and none of the two polymeric sleeves (100:50 or 150:50) affected the burst pressure of the dual-component graft significantly, compared to the native control or to each other. The dual-component graft groups comprising the 100:50 polymer blend sleeve for all scaffold decellularisation/sterilization combinations demonstrated a trend for increased burst pressures compared to all other groups and the native PTA control, but this increase was not statistically significant, except for the case of the M1-PAA-treated scaffold group, fitted with the 100:50 polymer blend sleeve, for which the increase was statistically significant compared to the M1-PAA-treated scaffold group without a sleeve (Figure 7G). These findings indicated that the dual-component graft can withstand similar burst pressures to the native PTA. However, further testing would be required to assess the dilation characteristics of the dual-component graft under physiological/pathological pressure conditions, with a view to assessing any potential aneurysmatic behaviour of the graft *in vivo*.

With regards to the antibacterial activity of the antibiotics-loaded sleeve and decellularized PTA, the sleeves that were fabricated with antibiotics supplemented in the electrospinning polymer blend solution (100:50A and 150:50A) were not able to suppress bacterial growth, most probably due to minimal release of the entrapped antibiotics, which, in turn, could have been due to the minimal degradation of these polymer blends over the 7-day period. Similar behavior was also observed with the sleeve samples that were soaked in antibiotic solutions for 1 h (100:50-5V/5G-1H and 150:50-5V/5G-1H). In this case, the antibiotic uptake by the polymers within 1 h, and the subsequent antibiotic release over 7 days, was not sufficient to suppress the growth of the strains tested. By increasing the incubation time to 24 h (100:50-5V/5G-24H and 150:50-5V/5G-24H), the polymers were able to suppress the growth of *S. Epidermis* and *S. Aureus*, but not *E. Coli*. The polymer samples that were incubated with 5 mg/mL VAN and 8 mg/mL GEN for 24 h (100:50-5V/8G-24H and 150:50-5V/8G-24H) demonstrated the best antibacterial activity, with both groups completely suppressing all three strains after 7 days. Overall, the 150:50-5V/8G-24H demonstrated the best performance, with a faster and complete antimicrobial suppression, which was also superior to the decellularized PTA group (Decel-PTA-5V/8G-24H) that could not achieve complete bacterial suppression after 7 days. The superior performance of the 150:50-5V/8G-24H group was confirmed in the disk diffusion assay, with this group demonstrating the largest inhibition area compared to the other two groups tested (100:50-5V/8G-24H and Decel-PTA-5V/8G-24H). The improved antimicrobial performance of the 150:50 polymer groups could be attributed to their structure, which demonstrated larger pores and thicker fibers that allowed higher impregnation by the antibiotics solutions and higher specific area for antibiotics binding, respectively, which in turn produced faster and increased release of antibiotics compared to the 100:50 sleeves.

## 5 Conclusion

This study assessed the biological, biomechanical, ultrastructural and antimicrobial performance of the components of a dual-component graft, comprising a decellularized PTA scaffold and an antibiotic-impregnated PCL/PEG blend sleeve. The inclusion of nuclease enzymes in the decellularization protocol reduced the amount of residual DNA in the scaffolds, as expected, without further affecting the other biological or biomechanical properties of the scaffold. Although the decellularization treatment was not able to effectively remove the α-gal xenoepitope, it could be used for the decellularization of human or α-gal-knockout small-caliber arterial grafts. Among the two sterilization treatments investigated, γ-irradiation with 150Gy produced the least detrimental effects, whereas treatment with 1% (v/v) PAA caused degradation of the collagen IV content of the scaffolds, and ultrastructural changes in the collagen and elastic fibers, and IEL. The assessment of the polymer sleeves, which were fabricated with two different PCL/PEG mass ratios, demonstrated that it is possible to tailor the biomechanical properties of the sleeve, so that it does not interfere with the dilation of the decellularized graft *in vivo*, or indeed to restrict any potential aneurysmatic behavior. Furthermore, the antimicrobial assessment demonstrated that it is also possible to tailor the antibiotics-releasing properties of the sleeve. Owing to the encouraging results obtained in this study, future work will focus on assessing the short- and long-term degradation rates of the PCL/PEG blend sleeve, as well as its antibiotics uptake and release kinetics, and any potential biofilm formation. In the case of the decellularized PTA scaffold, further studies will quantify potential detergent residues in the scaffold and will optimize the decellularization protocol in terms of more effective detergent and α-gal removal. Moreover, the biomechanical, haemodynamic and haemocompatibility function of the dual-component graft will be assessed *in vitro*, followed by *in vivo* testing in a large animal model.

## Data Availability

The raw data supporting the conclusion of this article will be made available by the authors, without undue reservation.
