# Supplementary figures and images for: Development of a dual-component infection-resistant arterial replacement for small-caliber reconstructions: A proof-of-concept study
Source: Front Bioeng Biotechnol. 2023 Jan 18;11:957458. doi: 10.3389/fbioe.2023.957458 (PMC9889865; doi:10.3389/fbioe.2023.957458)

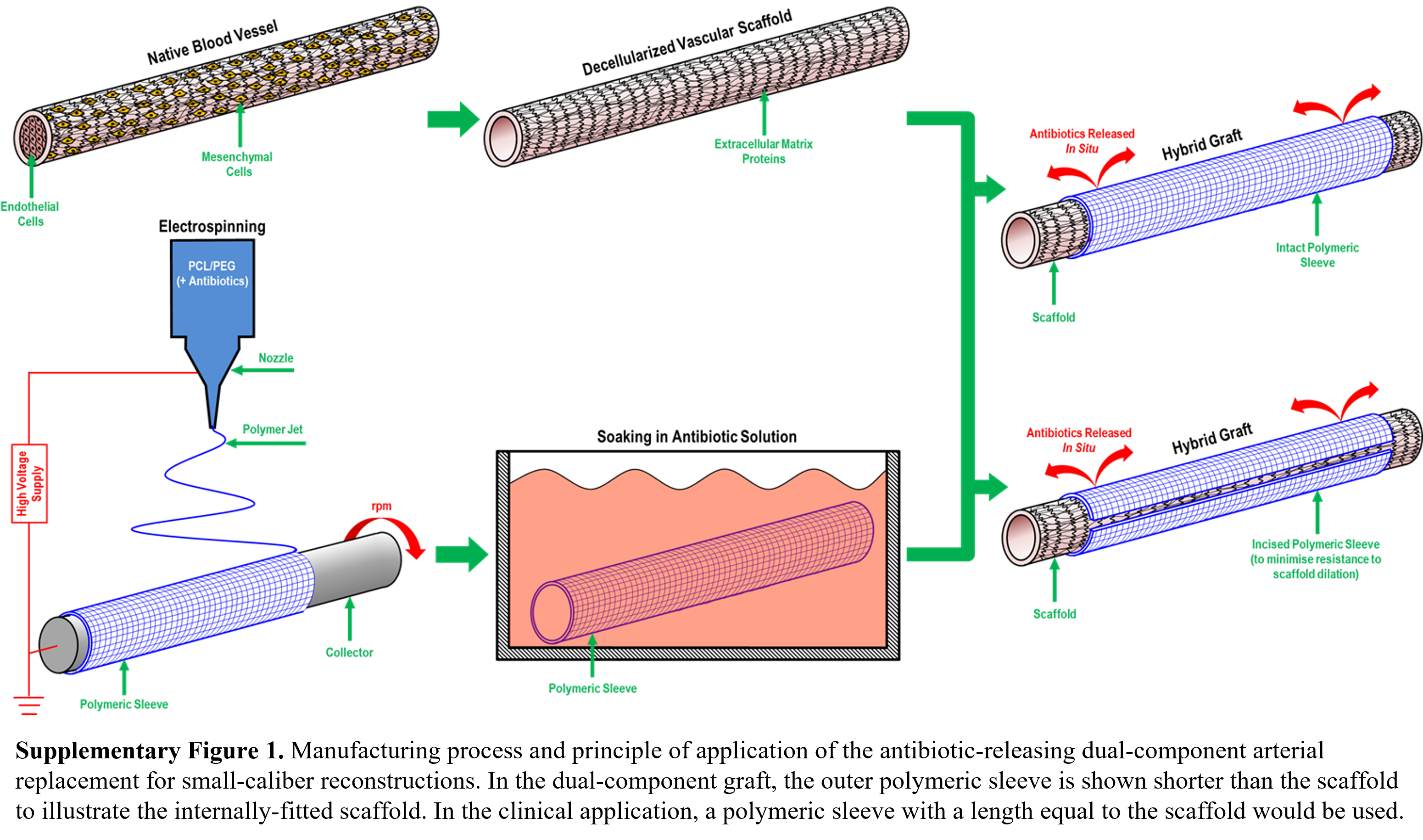

Supplement: Supplementary file 1 [file Image1.TIF]
